# *Trem2* deletion enhances tau dispersion and pathology through microglia exosomes

**DOI:** 10.1186/s13024-022-00562-8

**Published:** 2022-09-02

**Authors:** Bing Zhu, Yan Liu, Spring Hwang, Kailey Archuleta, Huijie Huang, Alex Campos, Rabi Murad, Juan Piña-Crespo, Huaxi Xu, Timothy Y. Huang

**Affiliations:** 1 Degenerative Diseases Program, Sanford Burnham Prebys Medical Discovery Institute, La Jolla, CA 92037 USA; 2 Proteomics Facility Core, Sanford Burnham Prebys Medical Discovery Institute, La Jolla, CA 92037 USA; 3 Bioinformatics Core, Sanford Burnham Prebys Medical Discovery Institute, La Jolla, CA 92037 USA; 4 Present address: Institute for Brain Science and Disease, Chongqing Medical University, Chongqing, China

**Keywords:** *Trem2*, Microglia, Exosomes, Tau pathology, Tau spreading, Alzheimer’s disease

## Abstract

**Background:**

Alzheimer’s disease (AD) is a neurodegenerative disorder that manifests sequential Aβ and tau brain pathology with age-dependent onset. Variants in the microglial immune receptor TREM2 are associated with enhanced risk of onset in sporadic Alzheimer’s disease (AD). While recent studies suggest TREM2 dysfunction can aggravate tau pathology, mechanisms underlying TREM2-dependent modulation of tau pathology remains elusive.

**Methods:**

Here, we characterized differences in progressive tau spreading from the medial entorhinal cortex (MEC) to the hippocampus in wildtype (WT) and *Trem2* knockout (KO) mice by injection of AAV-P301L tau into the MEC, and correlated changes in hippocampal tau histopathology with spatial and fear memory. We also compared effects of intraneuronal dispersion between cultured microglia and neurons using a microfluidic dispersion assay, analyzed differences in microglial tau trafficking following uptake, and quantified exosomal tau secretion and pathogenicity from purified WT and *Trem2* KO exosomes.

**Results:**

*Trem2* deletion in mice (*Trem2* KO) can enhance tau spreading from the medial entorhinal cortex (MEC) to the hippocampus, which coincides with impaired synaptic function and memory behavior. *Trem2* deletion in microglia enhances intraneuronal dispersion of tau in vitro between neuronal layers cultured in a microfluidic chamber, and the presence of exosome inhibitors can significantly reduce tau in exosomes and extracellular media from tau-loaded microglia. Although microglial *Trem2* deletion has no effect on tau uptake, *Trem2* deletion enhances distribution to endosomal and cellular pre-exosomal compartments following internalization. *Trem2* deletion has little effect on exosome size, however, proteomic analysis indicates that *Trem2* deletion can modulate changes in the microglial proteomic landscape with tau and LPS/ATP treatment conditions associated with exosome induction. Furthermore, exosomes from *Trem2* KO microglia show elevated tau levels, and feature enhanced tau-seeding capacity in a tau FRET reporter line compared to exosomes from WT microglia.

**Conclusion:**

Together, our results reveal a role for *Trem2* in suppressing exosomal tau pathogenicity, and demonstrates that *Trem2* deletion can enhance tau trafficking, distribution and seeding through microglial exosomes.

**Graphical abstract:**

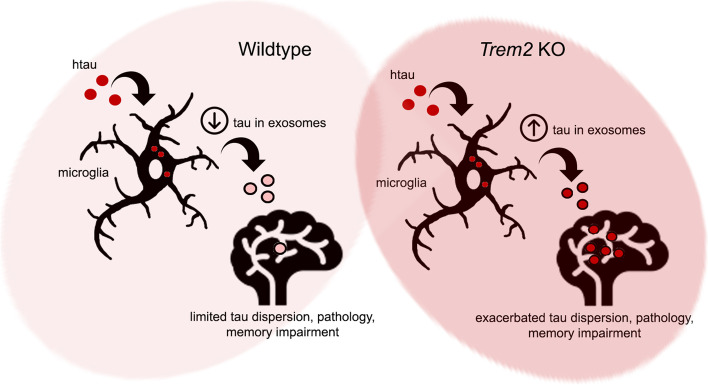

**Supplementary Information:**

The online version contains supplementary material available at 10.1186/s13024-022-00562-8.

## Background

Although symptoms of cognitive impairment and memory decline in Alzheimer’s disease (AD) ultimately manifests through neuronal dysfunction, AD is also obligately associated with neuroinflammation where glial cell types show chronic activation in AD brain [1, 23]. Recent identification of genetic variants linked to enhanced risk in sporadic AD onset through genome-wide association studies (GWAS) implicate numerous risk genes which feature enriched expression in microglia, including TREM2 and CD33 [9, 22, 25, 34, 50]. As an immune receptor almost exclusively expressed in microglia and myeloid cell types, mutational variants in TREM2 such as the rare R47H mutation has been shown to significantly increase risk of AD onset [22, 34], where TREM2 R47H has been shown to alter Aβ plaque morphology, microglial energy metabolism, and impaired binding to TREM2 ligands such as APOE and Aβ oligomers [3, 60, 65–67]. While a role for TREM2 in microglia has been described for microglial response to Aβ, whether TREM2 can similarly alter progression of tau pathology during AD onset is unclear. Recent studies suggest *Trem2* deletion (*Trem2* KO) can aggravate tau hyperphosphorylation and enhance morphological activation phenotypes in mouse models of tauopathy [7]. Further, *Trem2* KO or TREM2 R47H promote pathological tau seeding in an APP/PS1 AD mouse model [42]; *Trem2* deletion also aggravated tau spreading/pathology in a P301L tau/PS2 APP mouse model [41] as well as in 5xFAD brain injected with tau aggregates from human AD brain [21], together suggesting that in addition to Aβ [69], TREM2 dysfunction can also promote tau pathology. At this point, however, it is unclear how TREM2 can mediate tau dispersion and whether these mechanisms can impact tau-dependent impairment of cognitive function.

Given that TREM2 is expressed exclusively in microglia in brain, it seems likely that modulation of TREM2 may influence certain aspects of microglia behavior or function to consequently aggravate downstream tau pathology. Although microglia do not express tau, a potential role for microglia in altering tau pathology and spreading has been previously implicated in mouse models of AD [15]. Deletion of the murine fractalkine receptor CX3CR1 was previously shown to enhance microglial activation and reduce Aβ pathology in APP/PS1 mouse models [40], and was associated with enhanced tau pathology and memory [10]. Adoptive cell transfer of CX3CR1 knockout (KO) microglia could likewise induce tau pathology in hTau mouse brain [45], indicating that microglial activation could potentially aggravate tau pathogenesis. In agreement with this, recent evidence indicates that assembly of the NLRP3 inflammasome (comprising NLRP3, ASC and caspase-1) during microglia activation is required for tau hyperphosphorylation and aggregation [32]. Intriguingly, inflammasome activation is also induced through microglial deletion of an essential mediator of autophagosome biogenesis, Atg7, resulting in aggravated tau pathology and tau spreading in PS19 mouse brain [64]. Although enhancement of tau pathology is observed with NLRP3, CX3CR1 or microglial Atg7 deletion [10, 32, 45, 64], depletion of microglia in Tg4510 (P301L tau) mice at 12 months of age using the PLX3397 CSF1R inhibitor showed little effect on tau burden [8], despite upregulation of disease-associated microglia (DAM) gene signatures [8, 38, 39]. This suggests that microglia activation at early stages of tau pathology may be required to aggravate tau spreading and pathological accumulation.

Although growing evidence indicates that microglia can alter tau pathology, exact cellular mechanisms underlying microglia-dependent tau dispersion remain somewhat unclear. Previous studies demonstrate microglia can propagate pathological tau through exosomal extrusion/transmission [2]. Furthermore, pathological tau has been observed in microglia in PS19 (P301S tau) mouse brain [2], and tau seeds have been observed in microglia isolated from human AD brain, as well as in transgenic Tg4510 (P301L tau) mouse brain [27]. Microglia have also been shown to convert, package and release microvesicles containing Aβ species with enhanced toxicity [35]. Despite growing evidence that microglia exosomes can transduce proteotoxic protein species such as Aβ and tau, it remains unknown whether and how genetic components related to AD risk genes such as TREM2 and APOE can influence the dispersion and pathology of tau through exosome-associated mechanisms.

Herein, we observed that *Trem2* deletion enhanced transfer of human P301L tau from the medial entorhinal cortex (MEC) to the hippocampal dentate gyrus (DG) region in mouse brain, which correlated with increased tau pathology, reduced synaptic transmission and impaired spatial and fear memory. Using a microfluidic 3-chamber assay system, we show that *Trem2* KO microglia can transfer tau between isolated neuronal layers and demonstrate enhanced trafficking of internalized tau into pre-exosomal trafficking compartments, whereas treatment with exosome inhibitors suppressed extracellular tau extrusion in both WT and *Trem2* KO microglia. Purified exosomes from *Trem2* KO microglia exposed to tau oligomers also feature elevated levels of tau and show enhanced tau-seeding competency in vitro and induced enhanced pathological tau phosphorylation in WT mouse brain compared to exosomes from WT microglia. Together, these results implicate *Trem2* as a suppressor of pathological tau dispersion and demonstrate that *Trem2* deletion can aggravate pathological tau spreading through microglial extracellular exosomes.

## Results

### *Trem2* deletion exacerbates tau dispersion from the MEC to the hippocampal DG region

**Fig. 1:**
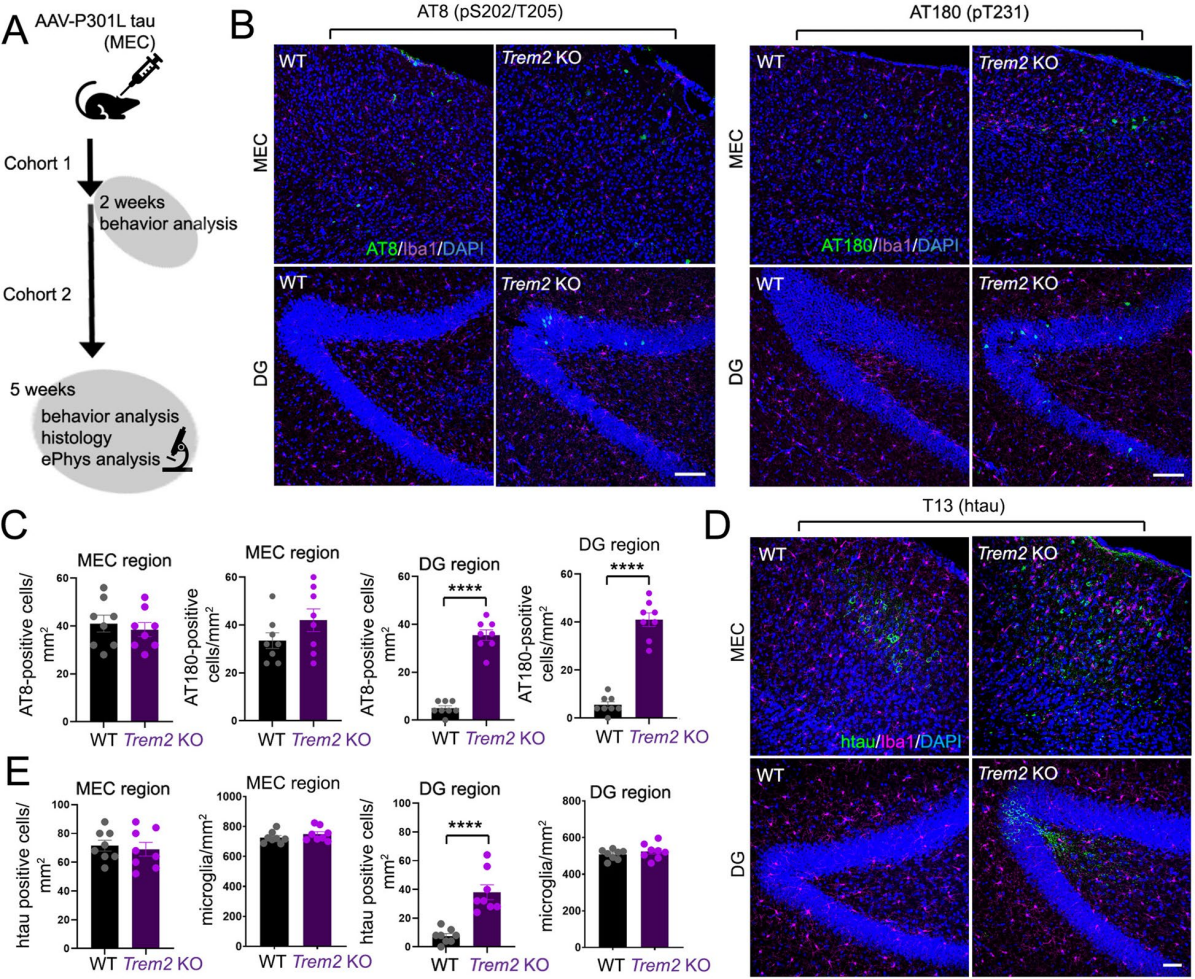
*Trem2* deletion aggravates pathogenic dispersion of tau pathology from the entorhinal cortex to the hippocampus. **A** Schematic depicting AAV-P301L tau (AAV-tau) injection and analysis. **B** AAV particles expressing human tau (AAV-P301L tau, “AAV-tau”) were stereotactically injected into the medial entorhinal cortex (MEC) of wildtype (WT) or *Trem2* KO mice at 4 months of age, and histological images from the MEC (upper panels) or hippocampal dentate gyrus (DG) (lower panels) were obtained by confocal microscopy 5 weeks post-injection. Representative images from the MEC were stained with AT8 (pS202, T205 tau) and AT180 (pT231 tau) antibodies (green), in addition to Iba1 (purple), and DAPI (blue) as indicated. Bar = 100 μm. **C** Quantification of AT8 and AT180-positive cells/mm^2^ in the MEC region or DG from WT and *Trem2* KO brain (*n* = 8 animals/genotype); graphs represent mean ± SE. Statistical significance was determined using unpaired Student’s t-tests (****, *p* < 0.0001). **D** Representative histological images in MEC and DG brain regions stained for human tau (T13, green), Iba1 (purple) and nuclei (DAPI, blue) in WT and *Trem2* KO mouse brain 5 weeks following AAV-tau injection. Bar = 50 μm. **E** Quantification of htau (T13)-positive cells/mm^2^ and number of Iba1-positive microglia in the MEC region or DG from WT and *Trem2* KO brain (*n* = 8 animal/genotype); graphs represent mean ± SE. Statistical significance was determined using unpaired Student’s t-tests (****p < 0.0001)

Previous studies indicate that microglial depletion can inhibit AAV-mediated tau propagation from the MEC to the DG region in mouse brain [2]. However, microglial components that modulate interneuronal tau dispersion have yet to be characterized. We therefore determined whether *Trem2* deletion could potentially affect tau dispersion from the MEC to DG. To this end, we stereotaxically injected AAV-P301L tau (“AAV-tau”) into the MEC region in 4-month-old wildtype (WT) and *Trem2* KO mice, and stained for pathological pS202/pT205 (AT8 antibody) and pT231 (AT180) tau phosphoforms 5 weeks following AAV-tau injection (Fig. 1A). Although we observed intense AT8 and AT180 staining in the MEC region at 5 weeks following AAV-tau injection in both WT and *Trem2* KO animals, we only observed robust AT8 or AT180 staining within the hippocampal DG region in *Trem2* KO animals, with little or no phospho-tau staining in WT DG (Fig. 1B,C and S1A). We also observed enhanced transmission of human tau in the DG area 5 weeks following AAV-tau injection in the MEC in *Trem2* KO animals compared to WT (T13 staining, Fig. 1D, E), indicating that pathological MEC/DG tau (p-tau) dispersion is likely triggered by transduced human P301L tau in the DG rather than pathological tau originating from the MEC. We observed no difference in microglia number in MEC and DG regions 5 weeks following MEC AAV-tau injection (Fig. 1E).

To determine whether tau pathology (p-tau) coincided exclusively with AAV-transduced transsynaptically from the MEC to DG through the perforant pathway, we stereotaxically injected AAV-GFP/AAV-tau together in the MEC of WT animals, and examined GFP and p-tau 5 weeks following injection (Fig. S1B). Similar to previous reports, we observe some convergence of GFP fluorescence/p-tau (AT8) in the MEC with AAV-GFP/AAV-tau injection, as well as GFP fluorescence in the outer molecular layer derived from EC axons 5 weeks following MEC injection (Fig. S1B) [2]. Although we observed AT8 p-tau staining in granule cells within the DG, we observed no GFP fluorescence in the granule cell layer (Fig. S1B), indicating dispersion of tau pathology was mediated through tau transfer rather than transsynaptic transport of the AAV vector. Thus, similar to previous studies utilizing this AAV-tau dispersion model [2], tau pathology in granule cells within the DG region is likely derived from pathological tau transmission rather than transsynaptic AAV transduction from axonal projections from the EC.

**Fig. 2:**
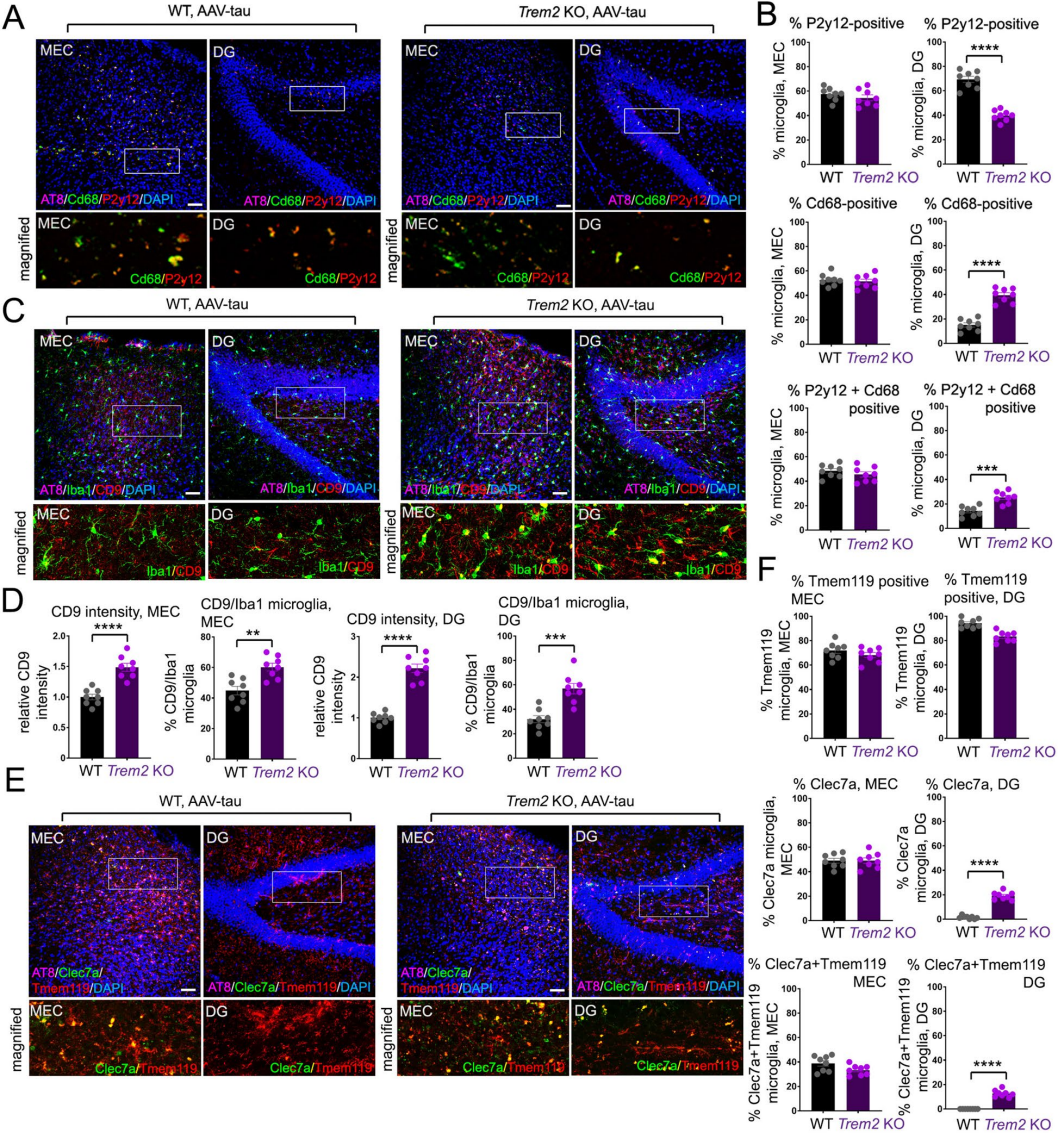
Characterizing microglial activation with MEC AAV-tau transduction. **A** MEC and DG regions from WT or *Trem2* KO mouse brain 5 weeks following AAV-tau injection. Sections were stained for AT8 (originally green, shown in purple), Cd68 (originally purple, shown in green), P2y12 (red) and nuclei (DAPI, blue), magnified regions are shown for Cd68/P2y12 colocalization. Bar = 50 μm. **B** Quantification of percentage P2y12 (top graphs), Cd68 (middle graphs) or P2y12/Cd68 double-positive microglia (bottom graphs) in the MEC region (left graphs) or DG (right graphs) from WT and *Trem2* KO brain (*n* = 8 animal/genotype). **C** MEC and DG regions from WT or *Trem2* KO mouse brain 5 weeks following AAV-tau injection. Sections were stained for AT8 (originally green, shown in purple), Iba1 (originally purple, shown in green), CD9 (red) and nuclei (DAPI, blue), magnified regions are shown for Iba1/CD9 colocalization. Bar = 50 μm. **D** Quantification of relative CD9 intensity (compared to WT, set to 1.0), or % CD9/Iba1 double-positive microglia in MEC or DG regions as indicated from WT and *Trem2* KO brain (*n* = 8 animal/genotype). **E** MEC and DG regions from WT or *Trem2* KO mouse brain 5 weeks following AAV-tau injection. Sections were stained for AT8 (originally green, shown in purple), Clec7a (originally purple, shown in green), Tmem119 (red) and nuclei (DAPI, blue), magnified regions are shown for Clec7a and Tmem119 colocalization. Bar = 50 μm. **F** Quantification of % Tmem119 positive (top graphs), Clec7a-positive (middle graphs), or Tmem119/Clec7a double positive (bottom graphs) microglia in MEC or DG regions as indicated from WT and *Trem2* KO brain (*n* = 8 animal/genotype). In (**B, D and F**), graphs represent mean ± SE from WT and *Trem2* KO brain (*n* = 8 animal/genotype). Statistical significance was determined using unpaired Student’s t-tests (**p < 0.01, ***p < 0.001, **** *p* < 0.0001)

We also characterized microglial activation in MEC and DG regions from WT and *Trem2* KO animals 5 weeks following AAV-tau MEC injection by staining for markers signifying microglial activation (CD68) and homeostatic (P2Y12) states (Fig. 2A). A mixture of CD68-positive, P2Y12-positive microglia, as well as CD68/P2Y12 double-positive microglia were observed in both WT and *Trem2* KO MEC (Fig. 2A-C). However, P2Y12-positive microglia were primarily observed in WT DG, whereas a mixture of CD68, P2Y12, and CD68/P2Y12 double-positive microglia were observed in Trem2 KO DG (Fig. 2A-C), indicating that microglia activation correlated with pathological tau states in MEC and DG regions, and microglial activation in DG correlated with tau dispersion in *Trem2* KO mouse brain. We also characterized expression of other genes expressed in homeostatic microglia such as Tmem119 or upregulated in disease-associated microglia (DAM) [38] or neurodegenerative microglia (MGnD) [39] such as CD9 and Clec7a. We observed that CD9 staining intensity, as well as the number of CD9/Iba1 positive microglia was significantly increased in both MEC and DG in *Trem2* KO mice compared to WT, 5 weeks following AAV-tau injection in the MEC (Fig. 2C, D). We observed no differences in Tmem119 positive microglia in MEC and DG regions, or in Clec7a or Clec7a/Tmem119 double positive microglia in the MEC of AAV-tau injected WT and *Trem2* KO animals; however, we observed increased Clec7a positive cells in DG, as well as Clec7a/Tmem119 double positive microglia in DG in *Trem2* KO animals compared to WT (Fig. 2E, F).

Together, these results suggest that *Trem2* deletion in mouse brain potentially aggravates dispersion of focally-administered tau at the MEC to the DG within a 5-week timeframe, where dispersion of tau pathology into the DG region coincides with the induction of DAM/MGnD associated markers such as CD68, CD9, and Clec7a.

### *Trem2* deletion potentiates cognitive behavior impairment and synaptic defects with tau dispersion from the MEC

**Fig. 3:**
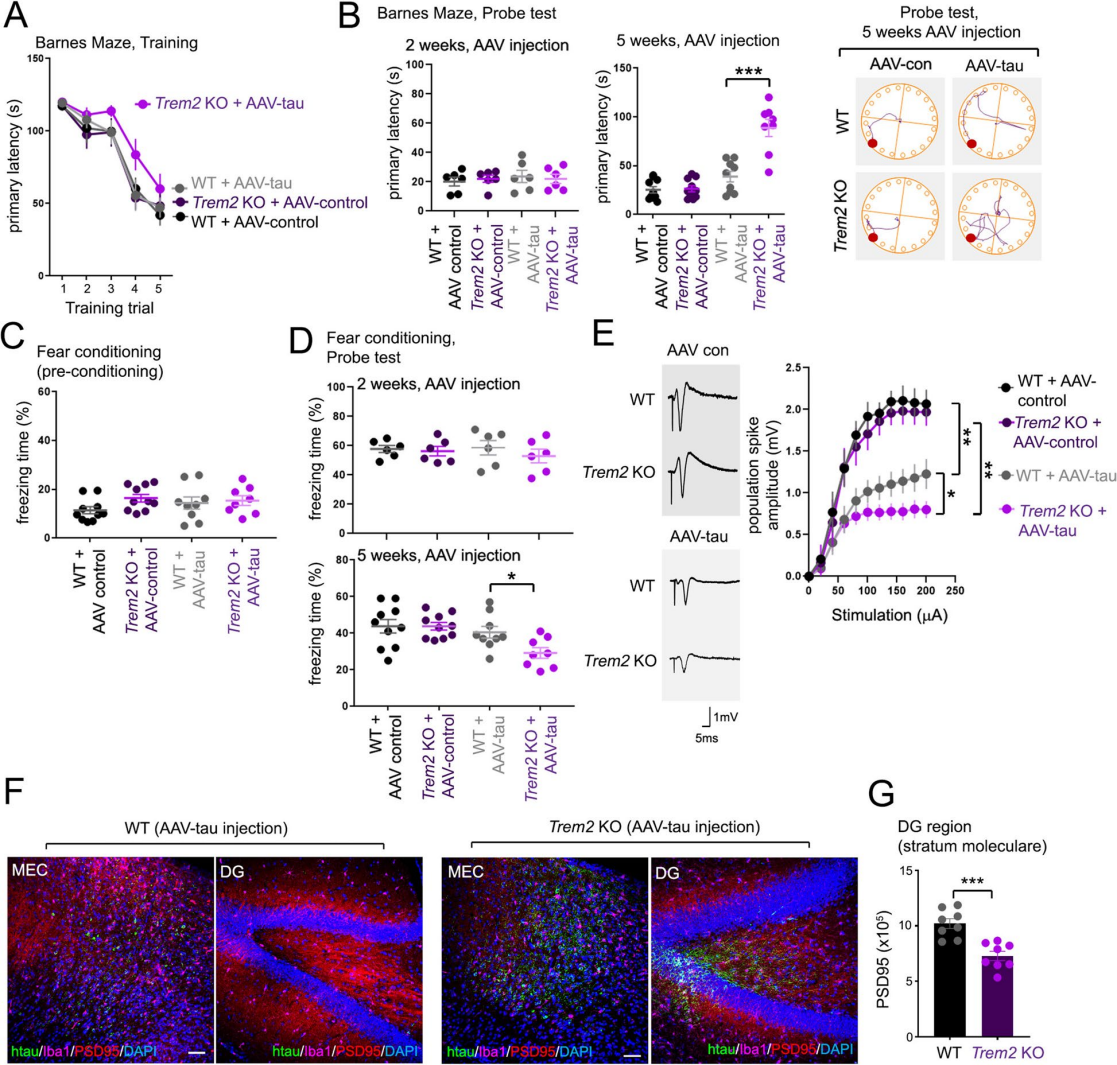
*Trem2* deletion aggravates memory and synaptic impairment associated with AAV-tau expression. **A-B** *Trem2* deletion aggravates tau-mediated spatial memory impairment. WT or *Trem2* KO animals were stereotactically injected with AAV control (AAV9-synapsin GFP) or AAV-tau constructs into the MEC region at 4 months of age, and subjected to 5 days of navigational training using the Barnes land maze (**A**); training of a cohort 5-weeks following AAV injection is shown. **B** 2 days following last training, spatial memory was evaluated by probe test in cohorts 2 weeks (left graph, *n* = 6 animals per genotype/treatment) or 5 weeks (right graph, WT/AAV con – *n* = 10; *Trem2* KO/con – *n* = 10; WT/AAV tau – *n* = 9; *Trem2* KO/AAV-tau – *n* = 8 animals) after AAV injection. Representative traces of motion tracks for WT and *Trem2* KO animals 5 weeks after AAV injection are shown; red circle indicates the target hole (traces, right panel). **C-D** *Trem2* deletion aggravates tau-mediated contextual fear memory impairment. WT or *Trem2* KO animals were stereotactically injected with AAV control or AAV-tau constructs as in (**A**), and characterized for freezing time during pre-conditioning 5 weeks after AAV injection (**C**), and in fear conditioning probe tests 2 weeks (top, *n* = 6 animals per genotype/treatment) or 5 weeks (bottom, WT/AAV con – *n* = 10; *Trem2* KO/con – *n* = 10; WT/AAV tau – *n* = 9; *Trem2* KO/tau – *n* = 8 animals) after AAV injection (**D**). **E** Population spike amplitude recorded in the DG region from acute hippocampal slices in WT and *Trem2* KO animals 5 weeks following AAV injection; population spike amplitudes are shown (WT control, 12 slices from *n* = 4 animals; WT AAV-tau, 13 slices from *n* = 4 animals; *Trem2* KO control, 15 slices from *n* = 5 animals; *Trem2* KO AAV-tau, 15 slices from *n* = 5 animals). Representative traces on the left depict depolarizing spikes following stimulation in WT and *Trem2* KO hippocampus as indicated. Graphs depict mean ± SE (error bars in (**E**) represent SE from total slice recordings from each experimental group), statistical significance was determined using One-way ANOVA with Dunn’s multiple comparison (**D**) and Two-way ANOVA (**B, E**) (*p < 0.05, **p < 0.01). **F** Representative histological images in MEC and DG brain regions stained for human tau (T13, green), PSD95 (red), Iba1 (purple) and nuclei (DAPI, blue) in WT and *Trem2* KO mouse brain 5 weeks following AAV-tau injection. Bar = 50 μm. **G** Quantification of PSD95 particles in DG from WT and *Trem2* KO brain (*n* = 8 animal/genotype) in (**F**); graphs represent mean ± SE. Statistical significance was determined using unpaired Student’s t-tests (***p < 0.001)

Given our observations that *Trem2* deletion can enhance tau dispersion from the MEC to DG following AAV-injection, we hypothesized that infiltration and presence of pathological tau indicators in hippocampus from *Trem2* KO animals may contribute to changes in memory and neuronal function. To test this, we used the dry-land based Barnes maze test originally designed for rats [6] and adapted for mice [5] to assess changes in spatial learning and memory, and contextual fear memory which requires coordinated activity between the hippocampus and amygdala [20, 51, 53], and compared behavioral differences in WT and *Trem2* KO mice injected with control AAV or AAV-tau (MEC region) 2 and 5 weeks following injection. During training trials, we observed impaired navigation to the target escape hole only in *Trem2* KO/AAV-tau injected animals at 5 weeks after AAV injection. While we observed little or no differences in navigation to the target escape hole at 2 weeks following AAV injection in WT or *Trem2* KO animals, *Trem2* deletion exacerbated memory impairment behavior in finding the target hole with AAV-tau injection compared to WT animals (Fig. 3A,B). We also observed slight, non-significant memory impairment in AAV-tau injected WT animals at 5-weeks post-injection compared to WT or *Trem2* KO AAV-control animals in Barnes Maze probe tests (Fig. 3B, right graph). Similar effects were also observed in AAV control/AAV-tau injected WT and *Trem2* KO mice in contextual fear conditioning experiments. Little or no difference in freezing behavior was observed in AAV control/AAV-tau-injected WT or *Trem2* KO animals in pre-conditioning experiments 5 weeks after injection (Fig. 3C), or in probe tests 2 weeks after AAV injection (Fig. 3D, top graph). However, we observed significant reduction in freezing time in AAV-tau/*Trem2* KO animals 5 weeks after AAV injection (Fig. 3D, bottom graph). Together, these results indicate that *Trem2* deletion manifests memory defects with AAV-tau injection in the MEC at 5 weeks after injection, which coincides with the appearance of phosphorylated tau in the hippocampus.

Given that *Trem2* deletion can impair memory with AAV-tau injection and dispersion from the MEC, we determined whether hippocampal memory impairment also coincides with defects in synaptic function. To this end, we recorded evoked local synaptic field potentials in the DG by stimulating the perforant path in acute hippocampal slices from WT and *Trem2* KO animals 5 weeks after MEC AAV-control or AAV-tau injection. In applying stepwise stimulation ranging from 0 to 200 μA to the perforant pathway and measuring population spike responses in dentate granule cells, we observed little or no difference in population spike amplitude in WT or *Trem2* KO animals injected with control AAV (Fig. 3E). However, significant reduction in population spike amplitude was observed with WT AAV-tau injection compared to AAV control injection in WT and *Trem2* KO animals (Fig. 3E). Moreover, AAV-tau injection in *Trem2* KO animals resulted in significantly impaired population spike responses compared to WT AAV-tau, or AAV-con injection in WT or *Trem2* KO animals (Fig. 3E).

We also determined whether pathological tau dispersion to the DG in *Trem2* KO brain could also impact synaptic markers such as PSD95 (Fig. 3F). We observed a reduced number of PSD95 puncta in the stratum moleculare in *Trem2* KO DG compared to WT 5 weeks following MEC AAV-tau injection (Fig. 3F, G). Together, these results demonstrate that *Trem2* deletion manifests hippocampal memory and synaptic defects which correlates with AAV-tau dispersion from the MEC to the hippocampus.

### *Trem2* deletion enhances microglia-mediated interneuronal tau dispersion in vitro

**Fig. 4:**
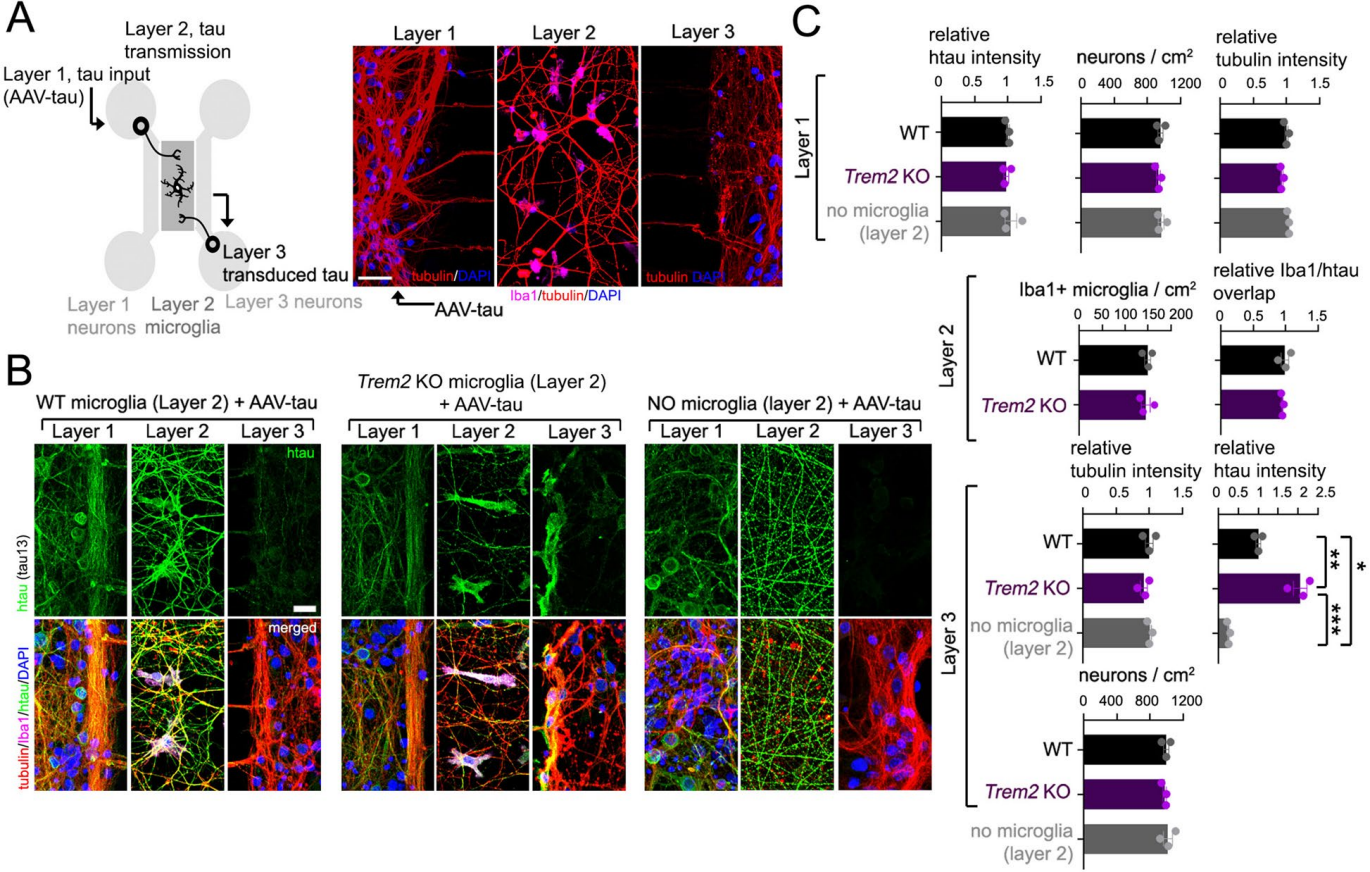
*Trem2* deletion enhances interneuronal tau transduction. **A** Schematic representation of a microglia-mediated interneuronal tau transduction system using a microfluidic 3-chamber culture system (left); a representative 3-chamber culture setup is shown (right). WT murine neurons are cultured in first and 3rd layers (Layers 1 and 3) (tubulin, red), and WT or *Trem2* KO microglia (Iba1, purple) are cultured in the intermediary chamber (Layer 2). AAV-P301L tau is transduced in the first layer, and effects of microglia in intermediary Layer 2 are determined on transmission of tau into neuronal Layer 3 by staining for human tau. **B** Neurons are exposed to 1 × 10^10^ VP/ml AAV-P301L tau in Layer 1, and transduction of P301L tau from Layer 1 into Layers 2 (microglia) and 3 (neurons) is quantified following transduction in the presence of WT or *Trem2* KO microglia, or without microglia in intermediary Layer 2 as indicated. Fluorescence imaging was used to characterize neuronal processes (tubulin, red), recombinant tau (h-tau, green), and microglia (Iba1). **C** Fluorescence intensity of human tau, tubulin, and neuronal numbers quantified in Layers 1 and 3, and quantification of microglia (Iba1) and Iba1/tau overlap in Layer 2. Graph depicts mean ± SE of 3 independent experiments (individual plots), averaged from 3 replicates per experiment. Statistical significance was determined by One-way ANOVA, with Tukey’s multiple comparison (*p < 0.05, **p < 0.01, ***p < 0.001)

Although our results so far indicate that *Trem2* deletion can promote tau-dependent dispersion and neuronal dysfunction, whether these effects are derived from alterations in microglia function remain unclear. We therefore reconstituted tau transfer between non-adjoining neuronal populations in vitro using a 3-chamber microfluidic assay system (Fig. 4A). This assay system comprises cultured primary neurons in the first and third layers, and primary microglia within the second layer; Layer 1 neurons are infected with AAV-tau (“tau input”), whereby tau is potentially taken up and transduced by microglia in the second layer (“tau transmission”) and consequently transmitted to Layer 3 neurons (“tau transduction”) (Fig. 4A). We therefore compared transduction of AAV-tau in Layer 1 to Layer 3 by WT or *Trem2* KO microglia, or in the absence of microglia in intermediary Layer 2 by fluorescence imaging (Fig. 4B). In the absence of microglia in Layer 2, we observed almost no transmission of tau into Layer 3 neurons (Fig. 4B,C); similarly, we observed weak transduction of tau into Layer 3 neurons with WT microglia cultured in Layer 2 (Fig. 4B,C). In contrast, *Trem2* KO microglia in Layer 2 enhanced transduction of tau between Layers 1 and 3 (Fig. 4B,C), thus implicating a role for *Trem2* deletion in promoting microglia-dependent transmission of tau between non-adjoining neuronal populations.

### *Trem2* deletion promotes microglial tau trafficking and excretion in exosomes

**Fig. 5:**
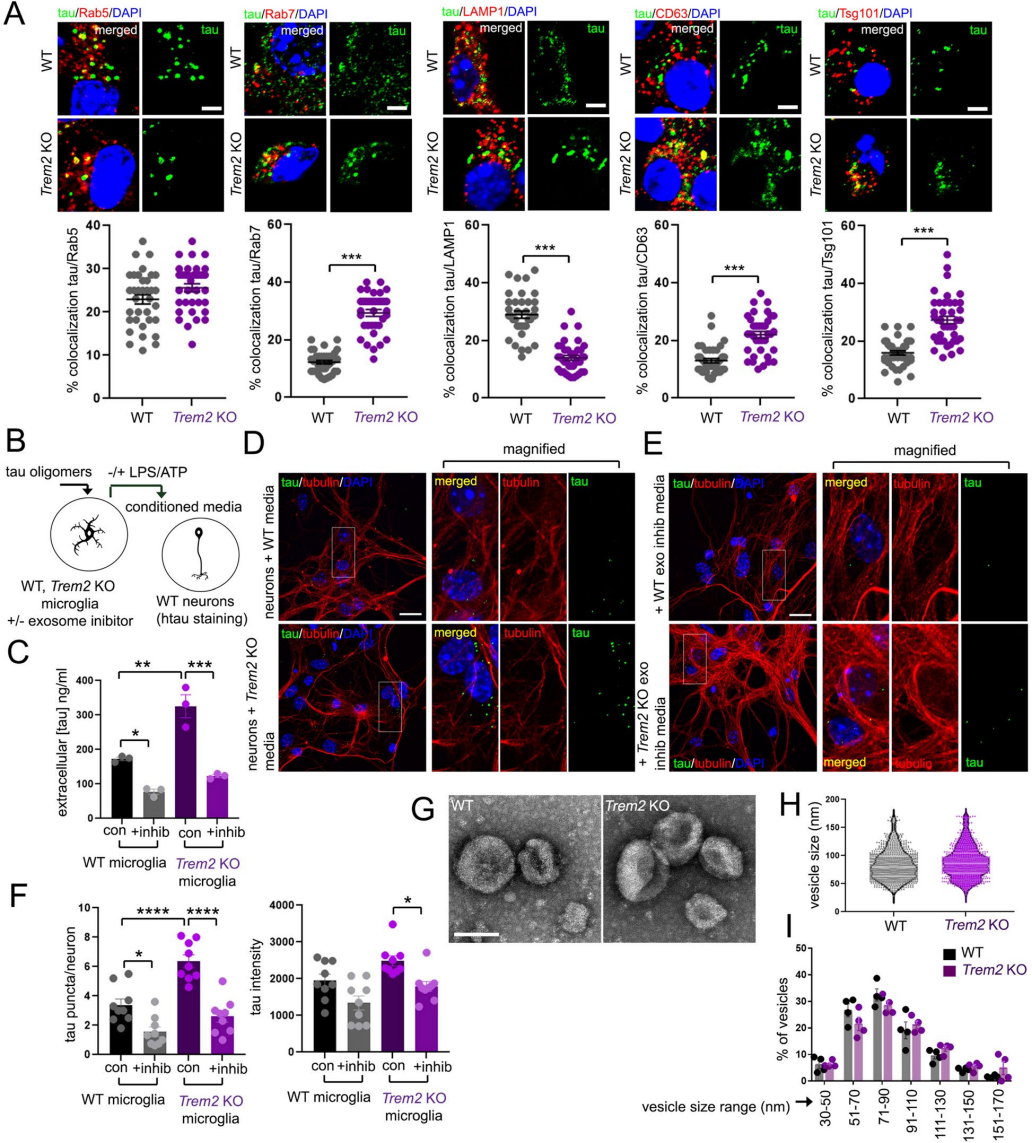
*Trem2* deletion promotes tau distribution to endosomal and pre-exosomal compartments, and promotes exosomal tau release. **A** *Trem2* deletion enhances endosomal tau sorting to pre-exosomal compartments. WT or *Trem2* KO microglia were incubated with 2.5 μg/ml tau oligomers for 24 h, and stained for endosomal (Rab5, Rab7), lysosomal (LAMP1), and markers for pre-exosomal vesicles (CD63, Tsg101) (red), and co-localization with human tau (green) was visualized by confocal microscopy. Region of overlap between intracellular markers (red) and tau (green) were determined as a proportion of total internalized tau in microglia the graphs shown (mean ± SE, where individual plots represent one cell from 3 independent experiments). Bar = 5 μm. Statistical significance was determined by unpaired Student’s t-test (***p < 0.001). **B** Experimental schematic: WT or *Trem2* KO microglia were loaded with 2.5 μg/ml tau oligomers for 24 h, washed with PBS and treated with 10 μM GW4869 for 3 h. Microglia were then treated with LPS/ATP and conditioned media was collected and subjected to tau quantification (ELISA) or neuronal tau uptake. **C** Exosomes were purified from conditioned media from tau-loaded WT or *Trem2* KO microglia in the presence (+inhib) or absence (con) of GW4869, and subjected to ELISA analysis to quantify exosomal tau released with LPS/ATP in conditioned media. **D-F** Quantifying tau uptake from microglia conditioned media in primary neurons. Primary WT mouse neurons were incubated for 48 h with conditioned media from tau-loaded WT or *Trem2* KO microglia untreated (**D**) or treated with GW4869 (**E**), and tau uptake was observed by confocal microscopy in cells stained for tau (T13, green) and tubulin (red); nuclei were visualized by DAPI staining (blue). Bar = 20 μm. **F** Quantification of tau puncta in neurons (left graph, tau puncta/neuron) or tau staining intensity (right graph, arbitrary units). Graphs represent mean ± SE, statistical significance was determined by One-way ANOVA with Tukey’s multiple comparison (*p < 0.05, **p < 0.01, ***p < 0.001, ****p < 0.0001). **G** Electron micrograph of exosomes prepared from WT or *Trem2* KO microglia. Bar = 100 nm. **H, I** *Trem2* deletion does not affect exosomal size. Exosomes purified from WT or *Trem2* KO microglia were plotted for exosomal vesicle size (**H**), as well as vesicle size distribution (**I**). Mean and quartiles are shown in violin plots in (**H**), graph in (**I**) represents mean ± SE

Given our results so far, *Trem2* deletion could potentially enhance tau uptake, thereby increasing microglia tau transduction through increased cellular tau loading and secretion. However, we observed no difference in tau loading in WT and *Trem2* KO microglia (Fig. 4B,C, “Iba1/tau overlap”), or in uptake of fluorescent tau oligomers by real-time live imaging or FACS analysis (Fig. S2A-C), suggesting that rates of tau uptake are relatively unperturbed with *Trem2* deletion. We also compared potential differences in directed intracellular trafficking of tau oligomers into various sorting compartments in WT and *Trem2* KO microglia by quantifying co-localization of tau with early endosomal (Rab5), late endosomal (Rab7), lysosomal (LAMP1) and multi-vesicular body (MVB)/exosome (CD63, Tsg101) markers by confocal imaging 24 h after exposure to tau oligomers (Fig. 5A and S2D-F). Although we see little difference in tau distribution in WT and *Trem2* KO microglia in Rab5, Rab7 and LAMP1 foci after 4 h, a clear shift in tau distribution to late endosomal Rab7 foci and away from LAMP1 compartments was observed in *Trem2* KO microglia at 24 h (Fig. 5A and S2E,F), indicating that *Trem2* deletion potentially enhances tau distribution to pre-exosomal compartments.

In addition to enhanced tau co-localization with Rab7 in *Trem2* KO microglia (Fig. 5A and S2E), we also observed enhanced tau co-localization with CD63 and Tsg101, and decreased co-localization with LAMP1 (Fig. 5A), implicating enhanced tau accumulation in multi-vesicular body (MVB) compartments/exosomal vesicles with *Trem2* deletion. To further determine whether exosome release is required for microglial-dependent tau transmission, we loaded WT and *Trem2* KO microglia with tau oligomers and stimulated exosome release with LPS/ATP in the presence or absence of exosome inhibitor GW4869 (10 μM) (Fig. 5B). Microglia conditioned media was collected, and subjected to tau quantification by ELISA analysis (Fig. 5C), or incubated with neurons and imaged for tau uptake by confocal microscopy (Fig. 5D, E). In the absence of exosome inhibitor, we found increased tau release in *Trem2* KO microglia compared to WT, and significant suppression of tau extrusion in microglia with GW4869 treatment (Fig. 5C). We also observed increased tau uptake in neurons incubated with conditioned media from tau-loaded *Trem2* KO microglia compared to WT (Fig. 5D,F), with significantly reduced neuronal tau uptake in neurons with conditioned media from tau-loaded WT and *Trem2* KO microglia pre-treated with GW4869 (Fig. 5E,F), suggesting that Trem2 deletion enhances intraneuronal tau transmissibility in a manner dependent on exosomes. To determine whether *Trem2* deletion could affect exosomal size or size distribution, we prepared exosomes from WT and *Trem2* KO microglia after induction with LPS/ATP as described previously [2], and observed little or no change in exosome size (Fig. 5G-H) or size distribution (Fig. 5I) in WT and *Trem2* KO exosomes by electron microscopy (EM) analysis. We also observed little or no cell death in WT or *Trem2* KO microglia with LPS/ATP treatment by TUNEL labeling/flow cytometry analysis (Fig. S3).

These results suggest that *Trem2* deletion promotes tau trafficking to late endosome/pre-exosomal compartments, and enhances extrusion of tau through exosome release without significantly altering exosomal size.

### Effects of *Trem2* deletion on exosomes, and cellular exosome and tau associated pathways in microglia

**Fig. 6:**
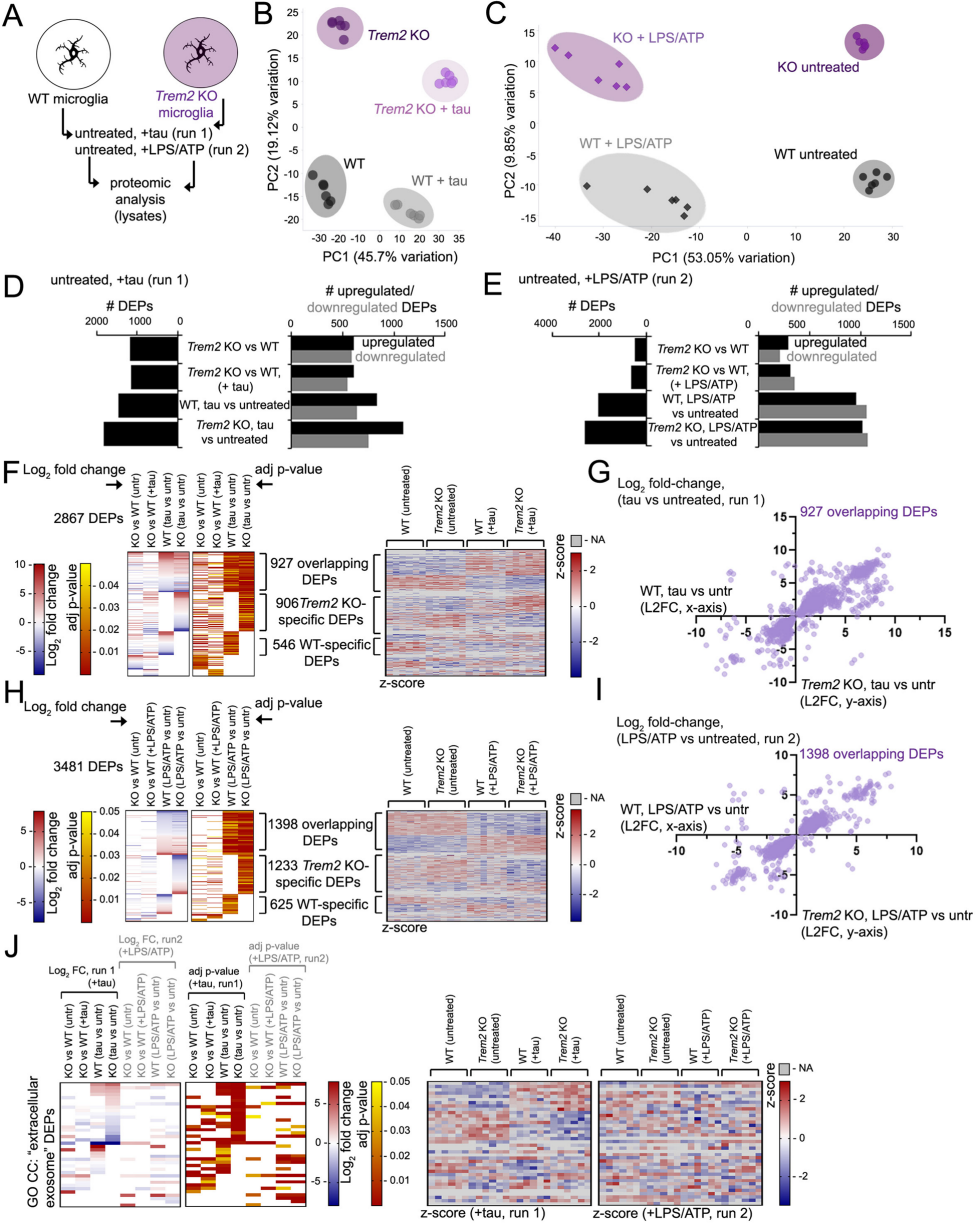
Characterization of proteomic profiles associated with exosome induction in *Trem2* KO microglia. **A** Schematic of the proteomic analysis scheme used; WT or *Trem2* KO microglia were left untreated or treated with tau oligomers (run 1) or LPS/ATP (run 2) and microglia lysates were subjected to proteomic analysis by label-free mass spectrometry. **B, C** PCA plots depicting distribution and clustering of proteomic profiles from WT and *Trem2* KO (“KO”) microglia untreated or exposed to tau oligomers (**B**) or LPS/ATP (**C**). PCA distribution of 6 individual replicate samples for each treatment group are shown. **D** Total (left graph) and upregulated (black)/downregulated (gray) (right graph) differentially expressed proteins (DEPs) in microglial cell lysates in four comparative analyses indicated (run 1): *Trem2* KO vs WT (both untreated), *Trem2* KO vs WT (both +tau), WT – tau vs untreated, and *Trem2* KO – tau vs untreated. **E** Total (left graph) and upregulated (black)/downregulated (gray) (right graph) DEPs in microglial cell lysates in four comparative analyses indicated (run 2): *Trem2* KO vs WT (both untreated), *Trem2* KO vs WT (both +LPS/ATP), WT – LPS/ATP vs untreated, and *Trem2* KO – LPS/ATP vs untreated. **F-I** Heatmap distribution and scatterplot distribution of overlapping DEPs in WT (x-axis) and *Trem2* KO (“KO”) (y-axis) microglia with tau (run 1) and LPS/ATP (run 2) treatment. **F** Heatmap showing Log_2_ fold-change and adj *p*-value of 2867 DEPs in WT and KO microglia with tau treatment; adjacent heatmap depicts z-score values of the genes listed on the left heatmap for individual samples (*n* = 6) from WT (untreated), *Trem2* KO (untreated), WT (+tau) or *Trem2* KO (+tau) microglia lysates as indicated; “NA” values derived from missing peptide values are indicated in gray. Log_2_ fold-change, z-score (red/blue) and p-value (red/yellow) scales are shown. **G** Scatterplot distribution of 927 overlapping DEPs identified in (**F**). **H** Log_2_ fold-change and adj p-value of 3481 DEPs in WT and KO microglia with LPS/ATP treatment; adjacent heatmap depicts z-score values of the genes listed on the left heatmap for individual samples (*n* = 6) from WT (untreated), *Trem2* KO (untreated), WT (+LPS/ATP) or *Trem2* KO (+LPS/ATP) microglia lysates as indicated; “NA” values derived from missing peptide values are indicated in gray. Log_2_ fold-change, z-score (red/blue) and p-value (red/yellow) scales are shown. **I** Scatterplot distribution of 1398 overlapping DEPs identified in (**H**). **J** Heatmap distribution, Log_2_ fold-change of GO CC “extracellular exosome” DEPs from combined runs 1 (tau) and 2 (LPS/ATP) from the 8 pairwise comparisons indicated. Z-scores of 6 replicates from each run are shown on the right, “NA” values derived from missing peptide values are indicated in gray

Given that microglia are pre-loaded with tau oligomers prior to induction of exosome release by LPS/ATP stimulation, we then determined whether *Trem2* deletion could affect cellular pathways related to tau or exosomes in response to tau or LPS/ATP exposure by proteomic analysis. To this end, we exposed WT or *Trem2* KO microglia to tau oligomers (run 1) or LPS/ATP (run 2), generated cell lysates from untreated, and tau or LPS/ATP-treated microglia, and subjected proteins to label-free proteomic analysis by mass spectrometry (Fig. 6A). We identified distinct proteomic profiles in WT and *Trem2* KO microglia both under steady-state and with tau oligomer or ATP/LPS treatment, with good consensus between replicate samples by Principle component analysis (PCA) (Fig. 6B,C). Although we identified a significant number of differentially-enriched or expressed proteins (DEPs) comparing proteomic profiles of *Trem2* KO (“KO”) to WT microglia under both steady state (1189 DEPs, run 1; 495 DEPs run 2) or with tau oligomer (KO tau vs WT tau, 1150 DEPs) or LPS/ATP treatment (KO LPS/ATP vs WT LPS/ATP, 660 DEPs), we observed dramatic changes in proteomic profiles comparing tau-treated to untreated WT (1473 DEPs) or *Trem2* KO microglia (1833 DEPs); we also observed robust changes with LPS/ATP treated to untreated WT (2023 DEPs) and *Trem2* KO (2631 DEPs) microglia (Fig. 6D,E; S4A,B and Table S1). Interestingly, we observed that while tau stimulation produced more upregulated compared to downregulated DEPs (Fig. 6D), LPS/ATP induction induced a roughly equivalent number of up and downregulated DEPs (Fig. 6E). Although we observed some variation in *Trem2* KO vs WT DEPs at steady-state between our two proteomic runs, we observed 64 significant DEPs in both runs with downregulation of Trem2 in *Trem2* KO lysates as expected (Fig. S4C).

In comparing tau or LPS/ATP-responsive DEPs in WT and *Trem2* KO microglia, we observed that although a proportion of tau- or LPS/ATP-responsive DEPs were shared between WT and KO, or between KO and WT microglia in response to tau or LPS/ATP, we also observed DEPs unique to WT and *Trem2* KO microglia at steady-state or with tau or LPS/ATP treatment (Fig. S4D, Table S1). We also observed changes in homeostatic or AD disease-associated microglial (DAM) genes differentially regulated in 5xFAD mouse brain [38] in cultured *Trem2* KO and WT microglia. *Trem2* KO microglia showed increased levels of homeostatic genes such as C1qa, C1qb and C1qc at steady-state in both datasets (runs 1 and 2), and KO also suppressed expression of induced DAM genes such Itgax and Lpl under both steady-state and tau or LPS/ATP treated conditions (Fig. S5A). Some differences in DAM response profiles were observed in cultured *Trem2* KO microglia with tau and LPS/ATP exposure by proteomic analysis; *Trem2* deletion downregulated homeostatic genes such as C1qb, C1qC, Ctss with tau, and was conversely upregulated in KO with LPS/ATP (Fig. S5A); induced DAM DEPs such as Lyz2, Ctsb, Ctsd, and Ctsz were suppressed with tau induction while induced with LPS/ATP in *Trem2* KO microglia (Fig. S5A). We also observed similar changes between tau and LPS/ATP stimulation in *Trem2* KO microglia, including upregulation of B2m, H2-D1, and downregulation of Csf1r. In general, we observed that *Trem2* KO deletion enhanced downregulation of various homeostatic (C1qa, C1qc, Ctss, Hexb) and DAM-associated DEPs (Lyz2, Ctsb*,* Cd68, Gusb, Ctsz) in response to tau compared to WT microglia; similarly many DAM genes induced with LPS/ATP in WT microglia (Apoe, Fth1, Itgax, Gusb) were unresponsive in *Trem2* KO microglia, overall suggesting that LPS/ATP and tau have both shared and varied effects on protein profiles which can be further modified by *Trem2* deletion.

To further compare similarities and differences in proteomic profiles with tau and LPS/ATP exposure, we identified overlapping DEPs with tau and LPS/ATP exposure in WT (Fig. S5B) and *Trem2* KO (Fig. S5C) microglia. We observed 302 and 473 shared DEPs in response to tau and LPS/ATP stimulation in WT and *Trem2* KO microglia respectively (Fig. S5B,C). Of these shared WT (302) and *Trem2* KO (473) tau, LPS/ATP-responsive DEPs, 141 common DEPs were shared (Fig. S6A); Gene Ontology (GO) analysis of these DEPs revealed enrichment of genes associated with “immune system process”, “inflammatory response” and “NFkB transcription factor activity” GO BP (Biological Process) categories, as well as “NFkB signaling pathway” GO KEGG categories (Fig. S6A, Table S2). Interestingly, microglia were shown to upregulate NFkB signaling in response to tau fibrils in vitro, as well as in PS19 tauopathy mouse brain to drive tau spreading and toxicity [62]. We observed some overlap between DEGs (Differentially Expressed Genes) from WT microglia exposed to tau fibrils [62] and WT or *Trem2* KO microglia exposed to tau oligomers (Fig. S6B). GO analysis of all DEPs (2867, run 1; 3481, run 2) (Fig. 6F,H) identified 52 DEPs associated with “mmu04064:NF-kappa B signaling pathway” KEGG pathways (Fig. S6C, Table S2). Although some NFkB-associated DEPs were exclusively observed in response to tau or LPS/ATP, upregulation of upstream NFkB regulators such as Il1b and Tnf shown to be induced by tau fibrils [62], as well as Nfkb2 were also upregulated in WT and KO microglia with both tau oligomers and LPS/ATP (Fig. S6C), indicating that tau and LPS/ATP potentially share some common microglia activation pathways such as NFkB that may be independent of *Trem2*.

GO analysis of tau (run 1) or LPS/ATP (run 2) proteomics datasets revealed a total of 38 GO CC (Cellular Component) “extracellular exosome” DEPs (Fig. 6J). *Trem2* KO microglia featured a more robust response compared to WT, with a more robust proportion of DEP response seen in KO, tau-treated microglia compared to KO, LPS/ATP-treated samples (Fig. 6J). A similar trend was observed in profiling proteins in exosomes from murine microglia from the ExoCarta database (exocarta.org) [37, 47, 48, 57] (Fig. S6D), with many DEPs altered with tau stimulation in KO microglia compared to other treatment groups.

We also observed downregulation of many endosomal PH-domain family sorting nexin (SNX), and intracellular trafficking vacuolar protein sorting (VPS) family members identified as DEPs in either or both WT and *Trem2* KO microglia with LPS/ATP stimulation with a lesser response with tau stimulation (Fig. S7A). Up to seventeen (17) SNX/VPS DEPs were downregulated in both WT and *Trem2* KO microglia with LPS/ATP stimulation, and 6 DEPs were specifically downregulated in *Trem2* KO microglia with LPS/ATP stimulation. Four SNX/VPS DEPs were downregulated specifically in WT microglia with LPS/ATP (2 upregulated) and not in KO with LPS/ATP, and fewer DEPs (such as Snx3, Vps37b, Snx15, Vps13c) were downregulated specifically with tau exposure. This suggests that *Trem2* deletion can modulate changes in trafficking/sorting components where many are downregulated with LPS/ATP stimulation.

We also determined whether tau or LPS/ATP treatment could alter expression of potential “tau protein binding” DEPs (GO MF (Molecular Function): 0048156) in WT and *Trem2* KO microglia (Fig. S7B, Table S2). Indeed we, observed downregulation of some putative tau binding proteins such as Bin1 in both tau and LPS/ATP (KO, tau or LPS/ATP vs untreated) groups; however, also observed differential upregulation of some potential interactors in KO-tau and KO-LPS/ATP groups (Fig. S7B). Altogether, our proteomic analyses indicate that tau and LPS/ATP conditions which stimulate exosomal release can induce robust proteomic changes in cultured microglia, and *Trem2* deletion can significantly alter proteomic shifts in response to tau and immunostimulatory/purinergic microglia activation; while some convergence on activating pathways such as NFkB were observed between tau and LPS/ATP treatment, *Trem2* deletion was found to alter exosome or DAM-associated components differently with tau or LPS/ATP stimulation.

### *Trem2* deletion enhances exosomal tau release, seeding and pathology

**Fig. 7:**
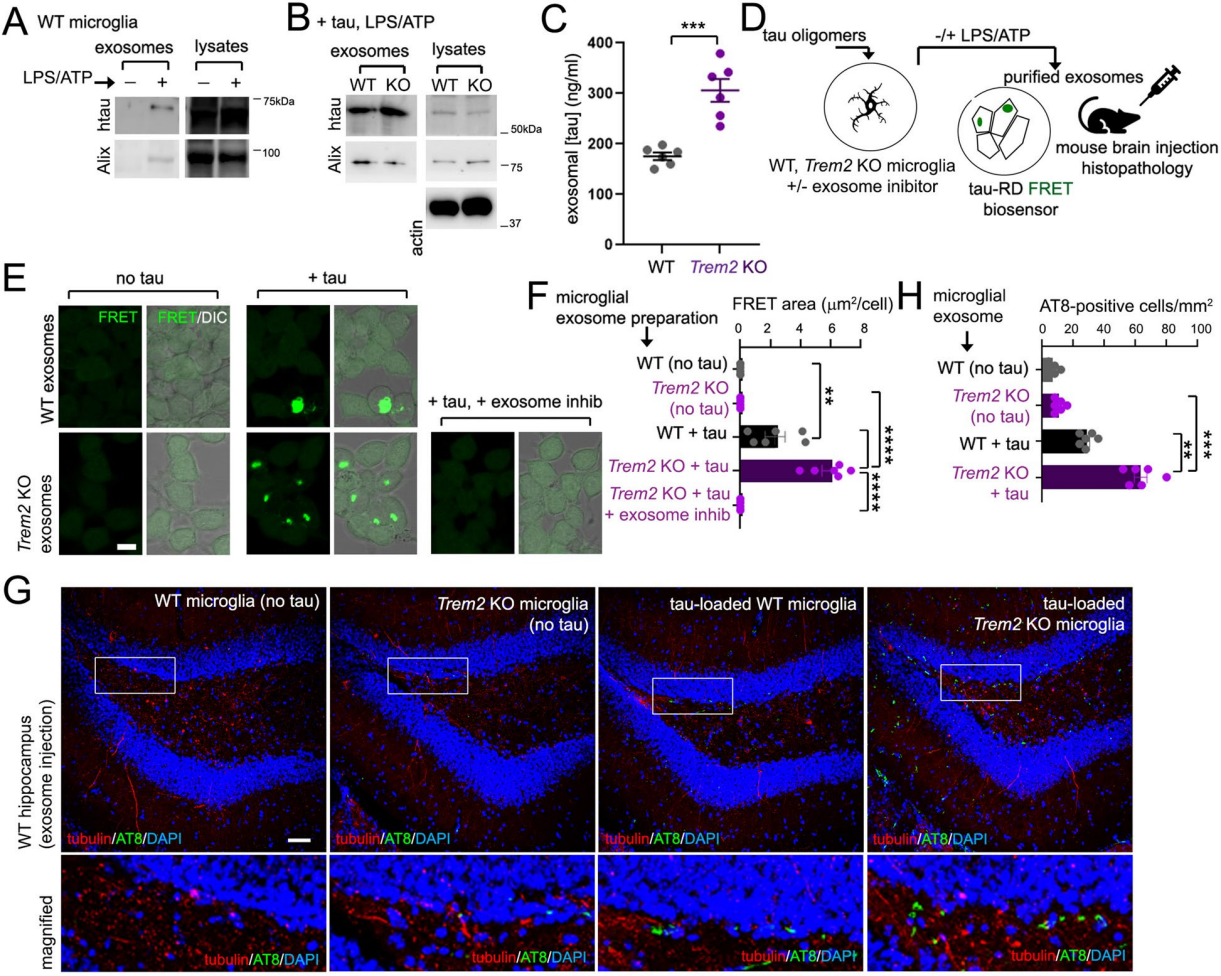
*Trem2* KO exosomes exhibit enhanced tau seeding activity. **A** Western blot of tau and Alix in lysates and exosomal preparations from tau-loaded WT microglia with or without LPS/ATP stimulation. **B** Comparison of tau and Alix in lysates and exosomes from tau-loaded WT and *Trem2* KO microglia following LPS/ATP stimulation by Western blot analysis. **C** ELISA measurement of tau levels in WT and *Trem2* KO microglial exosomes from 3 independent cultures, 2 replicate ELISA runs per culture. **D** Experimental design schematic, WT and *Trem2* KO microglia exposed to tau oligomers were treated (or untreated) with exosome inhibitor (GW4869), followed by exosomal induction with LPS/ATP. Purified exosomes were then assayed for tau seeding using the tau-RD FRET biosensor cell line, or injected into mouse brain for histopathological analysis. **E** Exosomes were purified from 1 × 10^7^ WT or *Trem2* KO microglia in the absence of tau (no tau), pre-loaded with tau oligomers (+ tau) and induced with LPS/ATP, or *Trem2* KO microglia pre-loaded with tau and pre-treated with 10 μM GW4869 prior to LPS/ATP release (+ tau, + exosome inhib), and applied to a tau RD reporter cell line. Tau fret signals were then visualized by fluorescence microscopy. Bar = 10 μm. **F** Quantification of the area of FRET-positive signals detected (μm^2^) per cell from representative images in (**E**). **G** Characterizing pathological effects of exosomal tau preparations in vivo. Exosomal preparations from tau-loaded or control (“no tau”) WT or *Trem2* KO microglia were stereotactically injected into 5-month old WT mouse hippocampus, and stained for AT8 pathology (green) or nuclei (DAPI, blue) 21 days after injection. Bar = 50 μm. **H** Number of AT8-positive cells (per 2.5 × 10^5^ μm^2^ area) were scored from 6 independently injected animals per treatment/genotype, graph represent mean ± SE. Graphs in (**C**), (**F**) and (**H**) depict mean ± SE. Statistical significance was determined by Student’s t-test (**C**), One-way ANOVA with Tukey’s multiple comparison (**F, H**), **p < 0.01, ***p < 0.001, ****p < 0.0001

To further characterize potential alterations in tau distribution and pathogenicity in *Trem2* KO microglia, we compared exosomal tau levels in *Trem2* KO and WT microglia following tau uptake and LPS/ATP induction by immunoblot and ELISA (Fig. 7A-C). As expected, we observed enrichment of exosome markers such as Alix, as well as tau in WT microglia exosome preparations with LPS/ATP stimulation with little or no Alix/tau present in exosome preparations without stimulation by immunoblot analysis (Fig. 7A). Comparison of tau levels in exosome preparations from WT and *Trem2* KO microglia by Western blot and ELISA revealed significantly increased tau levels in exosomes derived from *Trem2* KO microglia (Fig. 7B,C).

Next, we determined whether exosome preparations from WT and *Trem2* KO microglia could induce consequent tau seeding in an HEK293-derived tau FRET reporter line (tau-RD) [26], and examined effects of stereotactically injecting WT or *Trem2* KO exosome preparations in mouse brain (Fig. 7D). We therefore induced and purified exosomes with LPS/ATP in WT and *Trem2* KO microglia without tau-preincubation (“no tau”) or in presence of tau oligomers (“+ tau”), or *Trem2* KO microglia in the presence of tau and addition of an exosome inhibitor (GW4869) prior to LPS/ATP treatment (“+ tau, + exosome inhib”); a representative set of microglia was stained to confirm tau internalization (Fig. S7C). We then incubated tau-RD cells with exosome preparations and assayed for tau seeding by FRET imaging and analysis (Fig. 7E). We found that exosomes from *Trem2* KO microglia exposed to tau oligomers showed more tau FRET activity compared to exosomes prepared from WT microglia with tau oligomer treatment (Fig. 7E,F). No tau seeding activity was observed in tau RD cells in exosome preparations from WT or *Trem2* KO microglia in the absence of tau, or in the presence of tau with addition of GW4869 (Fig. 7E,F).

To determine whether exosomal preparations from tau-loaded or non-tau treated (control) WT and *Trem2* KO microglia could induce tau pathology in vivo, we stimulated WT and *Trem2* KO microglia in presence or absence of tau oligomers with LPS/ATP and stereotactically-injected purified exosomes from conditioned media into WT mouse hippocampus. Mice were sacrificed and histological mouse brain sections were stained for AT8 21 days after exosome injection (Fig. 7G). We observed no AT8-positive cells in the hippocampus of animals injected with exosomal preparations from non-tau loaded WT and *Trem2* KO microglia (“no tau”); however, we observed that while tau-loaded WT microglial exosomal preparations induced hippocampal AT8-ptau pathology to some degree, these effects were significantly enhanced through exposure to tau-loaded *Trem2* KO microglial exosomes (Fig. 7G,H). These results suggest that *Trem2* deletion can enhance microglia exosome pathways and increase transduction of seed-competent tau isoforms through exosomes.

Together, our results demonstrate a role for *Trem2* in suppressing exosomal tau dispersion in a manner at least partially dependent on downregulating tau distribution to exosomal vesicles.

## Discussion

Early studies characterizing the progression of human neurofibrillary pathology during AD progression noted distinctive spreading of tangles originating from the entorhinal region to isocortical regions at later stages of disease [11]. Interestingly, tau pathology appears to spread progressively along neuroanatomically interconnected regions within the brain [24, 56, 61], potentially through the dispersion of tau aggregates between synaptically adjoined neurons to promote seeded aggregation in unaffected downstream cells [16, 17, 19, 43]. Various mechanisms have been proposed to describe how intracellular tau aggregates are transferred between cells primarily through studies in vitro; potential means of intracellular tau transfer include cell to cell spreading through interconnected membrane nanotubes [59], vesicle-free mechanisms through tau secretion [36, 49], or unconventional pore-mediated tau extrusion [12]. Despite these observations, whether tau is exclusively transmitted between neurons and how transmission occurs remains somewhat unclear.

Interestingly, recent evidence indicates that microglia play a fundamental role in mediating tau spreading through tau extrusion and dispersion in extracellular exosomes [2], which may involve stimulation of exosome release through the activation of purinergic receptors such as P2RX7 [52]. Microglia feature a distinctive expression profile in AD mouse brain, and modulate a specific subset of “disease-associated” or “neurodegenerative” microglial (DAM, MGnD) transcripts [38, 39]. Tetraspanin components such as Cd9 previously shown to mediate exosomal biogenesis [13] are upregulated in microglia from 5xFAD mouse brain [38]; however, how age-related AD stress potentially affects microglia exosomal biogenesis remains yet unclear. Given that genomic studies have identified enrichment of microglia gene variants related to AD risk such as TREM2 [9, 22, 25, 34, 50], it seems likely that pathological events such as exosome dysregulation may be affected by altered function of TREM2 and other microglial AD risk variants.

Although our results revealed no difference in internalization of tau oligomers, we observed enhanced trafficking of tau to the late endosome and compartments containing the ESCRT-I component Tsg101 and the MVB-enriched tetraspanin, CD63, suggesting that *Trem2* deletion can enhance enrichment of certain cargo such as tau to exosome-destined compartments. Interestingly, *Trem2* deletion has been recently shown to alter exosomal content in macrophages, where increased levels of the micro RNA miR-106b-5p in macrophage-derived, *Trem2* KO exosomes induced mitochondrial impairment in hepatocytes [28]. Additionally, iPSC-derived microglia from heterozygous carriers of the AD-associated R47H allele also featured alterations in exosomal content, and exosomes derived from TREM2 R47H microglia showed reduced protection from H_2_O_2_ toxicity in SH-SY5Y cells [44]. Thus, growing evidence indicates that TREM2 dysfunction can alter exosomal content and potential downstream toxicity. At this point however, how *Trem2* deletion or dysfunction can influence exosome biogenesis or trafficking remains unclear. Interestingly, our proteomic analysis indicates that *Trem2* deletion can potentially alter proteins associated with exosomes, intracellular exosome biogenesis, and tau-binding with tau and LPS/ATP stimulation, suggesting that *Trem2* deletion may alter proteomic profiles linked to tau trafficking and extrusion pathways.

Although genetic perturbations associated with microglia activation such as deletion of CX3CR1, NRPL3 or Atg7 have been previously shown to aggravate tau pathology as discussed above [10, 32, 45, 64], the connection between microglia activation and tau appears to be complex. With respect to microglia activation, *Trem2* has been shown to be essential in the transition of microglia from homeostatic to late-stage DAM states in neurodegenerative mouse brain [38, 39]. Microglia are thought to transition between homeostatic and an early DAM state (stage 1 DAM) independent of *Trem2* which involves the downregulation of homeostatic genes such as *Cx3cr1* and the purinergic receptor *P2ry12* [38]. *Trem2*-dependent progression from early to late-stage DAM (stage 2 DAM) involves upregulation of various transitional markers (Lpl, Cst7, Cd9, Axl) which are associated with upregulation of various pathways linked to lysosomal, phagocytic and lipid metabolic function. Since *Trem2* deletion is anticipated to suppress microglia conversion from homeostatic to disease-associated states, one possibility is that cellular changes uncoupled to DAM or MGnD transcriptional signatures could account for enhanced tau spreading in *Trem2* KO microglia. In support of this, our proteomics analysis implicates alteration of exosome and tau-associated proteins in cultured *Trem2* KO microglia with tau or LPS/ATP stimulation that may enhance exosome-mediated tau spreading with *Trem2* deletion.

In agreement with our results indicating that *Trem2* deletion can aggravate tau dispersion, previous studies have shown that tau pathology and/or spreading is enhanced in most tau mouse model systems where *Trem2* is downregulated or deleted. For example, shRNA-mediated *Trem2* downregulation [33], *Trem2* haploinsufficiency [18, 55], *Trem2* deletion [7] in mouse tauopathy models, or *Trem2* deletion in 5xFAD mouse brain injected with tau derived from human AD brain (“AD-tau”) [21] can aggravate tau pathology which together, suggests that *Trem2* normally restrains pathological effects associated with tau. Interestingly, some differences with respect to *Trem2* haploinsufficiency (*Trem2* ^+/−^) and homozygous deletion (*Trem2* ^−/−^) have been observed with respect to tau pathology in mice; while *Trem2* haploinsufficiency enhances pathological ptau (AT8) [18, 55] and fibrillary tau forms [55], homozygous *Trem2* deletion potentially attenuates ptau and fibrillary tau in PS19 brain [55]. Although this indicates that homozygous *Trem2* deletion may have reduced pathogenicity compared to *Trem2* haploinsufficiency, we note that since tau pathology was quantified at late stages (8–9 months) in PS19/Trem2^+/−^ and PS19/Trem2^−/−^ mice [55], analysis at an advanced pathological age may mask potential effects on tau spreading which would occur during early stages of pathological tau accumulation. In support of this notion, depletion of microglia showed little or no effect on tau plaque burden in aged 12 month-old Tg4510 mouse brain [8], further suggesting that microglia alterations at early stages impact late stage pathology in mouse tauopathy models.

Additionally, pathological context may also manifest differences observed between *Trem2* haploinsufficiency and homozygous deletion; recent evidence indicates that homozygous *Trem2* deletion can affect tau pathology in a P301L tau, PS2APP (PS2 N141I; APP KM670/671NL) combined mouse line, with little effect in *Trem2* WT or haploinsufficient (het) animals [41]. Given that *Trem2* has been shown to bind directly to Aβ and alter microglia response [66, 67], it is tempting to speculate that *Trem2* haploinsufficiency can still limit tau pathogenesis in response to Aβ proteotoxicity. Aside from differences in tau pathogenesis with homozygous and heterozygous deletion, downregulation of *Trem2* function appears to have deleterious consequences in exacerbating tau pathology and spreading. As our results here only provide evidence that homozygous *Trem2* deletion can enhance tau spreading, future work may determine how *Trem2* levels can differentially affect global tau pathology (phosphorylation and conformation) and tau spreading, and its relationship to Aβ.

Although our results here and from other published studies implicate an active role for microglia activation in aggravating tauopathy, the question remains why microglia depletion in some instances, for example in 5xFAD mouse brain with AD-tau injection, can also aggravate tau pathology [21], while other studies that show reduction in tau pathology in PS19 mouse brain with microglia depletion [2]. One possibility may be that microglia-independent mechanisms of tau transduction may predominate differently in various combined Aβ/tau or tau-only mouse models, which may involve pathological effects between cell types. For example, direct transfer of tau between neurons has been demonstrated, and appears to be enhanced by neuronal stimulation [63], and recent evidence also indicates that tau transfer can also occur between neurons and astrocytes [46]. This suggests that in addition to contributions from microglia, tau transduction mechanisms may involve other cell types which may introduce additional effects in the presence of amyloid proteotoxicity.

## Conclusions

In summary, we provide pioneering evidence that *Trem2* deletion can enhance tau spreading from the MEC to DG in the hippocampus, which coincides with behavioral fear and memory deficits, and our results altogether suggests that *Trem2* function normally reduces tau trafficking to late endosomes/MVBs and exosomal vesicles in microglia to limit tau dispersion and seeding. Moreover, our results also indicate that *Trem2* deletion can affect intracellular trafficking of internalized cargo in microglia, suggesting that in addition to immune signaling, proper *Trem2* function may be important in intracellular trafficking pathways in microglia. As *Trem2* dysfunction has been previously shown to affect Aβ plaque pathology, these results also consolidate a dual role for *Trem2* in altering both Aβ and tau pathology and consequent neurodegenerative effects associated with Aβ and tau. Importantly, results presented here implicate a protective role for *Trem2* early in tau pathogenesis and dispersion where early intervention could potentially stem or delay global effects of tau-associated degeneration in AD and potentially other related tauopathy disorders.

## Methods

### Mouse models

*Trem2* knockout (KO) (Stock no. 027197) and Wild-type (WT) C57BL/6 J mouse strains were purchased from The Jackson Laboratory (JAX). Both KO and WT animals were maintained as homozygous lines. Postnatal day 2–3 WT and *Trem2* KO mice were used for dissection and generation of primary microglial cultures. Since hormonal effects are negligible at this stage, mixed primary microglial cultures were taken from both males and females. Animal procedures did not include additional drug or treatment regimen other than that described. Mouse lines were housed with littermates with free access to food and water under a 12 hour light/day cycle. All animal procedures, including husbandry were performed under the guidelines of the Institutional Animal Care and Use Committee at Sanford Burnham Prebys Medical Discovery Institute.

### Stereotaxic AAV injection

Control and recombinant AAV-P301L tau (AAV9) vectors have been described previously; AAV-GFP and AAV-P301L tau plasmid vectors were generously provided by Dr. Tsuneya Ikezu [2]. 1 × 10^9^ AAV particles were stereotaxically/bilaterally injected into the MEC (layer II/III) using the following coordinates: anteroposterior, − 4.75 mm; lateral, + 2.9; dorsoventral, − 4.6. Mice were then subjected to behavioral analysis, or histological staining 14 (behavior only) or 35 (histology and behavior) days following stereotaxic injection.

### Immunohistological staining and analysis

Mice stereotaxically injected with AAV particles were sacrificed for immunohistological analysis at 35 days post-injection. Mice were anesthetized with 4% isoflurane and intracardially perfused with PBS. Brain tissues were harvested and fixed in 4% paraformaldehyde at 4C for 24 h. Tissues were washed in PBS and cryoprotected in PBS containing 30% sucrose. Tissues were embedded in OCT containing 30% sucrose (at 1:1 v/v) and free-floating coronal brain cryostat sections (25 mm) were collected. Data were collected and analyzed in a double-blind fashion.

For detection of Iba1, ptau, and tau, brain slices were stained using antibodies as follows: Goat anti-Iba1 (1:400, ab5076, Abcam), AT8 (1:500, MN1020 ThermoFisher), AT180 (1:500, MN1040 ThermoFisher), T13 (1:500, #835201 Biolegend), PSD95 (1:200, #3450 s Cell Signaling), Cd68 (1:250, MCA341R BioRad), P2y12 (1:200, #69766 Cell Signaling Technology), Cd9 (1:100, 20,597-I-AP Proteintech), Tmem119 (1:150, #90840 Cell Signaling), Clec7a (1:100, Invivogen), GFP (1:250, NB600–308 Novus). Alexa Fluor 488, 568 or 647 secondary antibodies (1:400, ThermoFisher) were used, DAPI counterstains were applied to the sections, and image z stacks were acquired from multiple sections (up to 9) from each animal using a Zeiss LSM 710 laser-scanning confocal microscope.

Staining intensity of human tau in cultured neurons was quantified using Imaris image analysis software (Bitplane, Oxford Instruments), and phospho-tau epitopes quantified in histological sections were scored manually by identifying AT8 or AT180-positive cells in imaged MEC or DG regions. Quantification of PSD95 puncta in the stratum moleculare within the hippocampus was also performed using Imaris by defining a region of interest (ROI) defined immediately above of the granular cell layer (GCL). The number of PSD95 puncta in each ROI was measured using Imaris, and calculated using a set intensity threshold, expressed as number of PSD95 puncta/mm^2^. Percentage of P2y12, Cd68, Tmem119, Clec7a, CD9 positive microglia and P2y12/CD68, Tmem119/Clec7a, CD9/Iba1 double positive microglia was quantified using Imaris.

### Behavioral analysis

Barnes maze test was performed as described previously [4]. Briefly, WT or *Trem2* KO mice stereotaxically injected with AAV-tau or AAV control (AAV9-synapsin GFP) were habituated on day 1, where mice were placed in the center of the maze underneath a clear 3500-ml glass beaker for 30 s. Mice were slowly guided to the target hole leading to the escape cage by moving the glass beaker over the target hole within a span of 10–15 s. Mice were then given 3 min to enter the target hole, and gently forced to enter in the event no entry was apparent. Training was initiated the next day. Mice were placed inside an opaque cardboard cylinder, 10″ tall and 7″ in diameter, in the center of the Barnes maze for 15 s. The cylinder was then removed, and mice were allowed to explore the maze for 2 mins. Upon entering the escape cage, the mouse was allowed to remain in the escape cage for 1 min; otherwise, the mouse was gently guided to the escape hole using a glass beaker and allowed to freely enter the escape cage. In case the mouse did not enter the escape cage within 3 min, it was gently nudged with the beaker to enter. Five trials were performed during training, with 3 trials on day 1, and 2 trials on day 2. Forty-eight hours after the last training session, a probe test to analyze the behavioral characteristics of the mice seeking the target area was monitored using a digital camera controlled by the ANY-maze video tracking system (Stoelting Co.). Subsequent analyses of the probe test parameters were processed using ANY-maze software, where statistical analyses and significance values were calculated using GraphPad Prism (Dotmatics, Boston, MA).

Contextual fear conditioning behavior tests were performed using the Freeze Detector System (San Diego Instruments, CA). Twenty-four hours before training initiated, mice were placed in the conditioning chamber and allowed to freely explore the chamber for 5 mins. On the first day of training, mice were placed in the conditioning chamber and allowed to freely explore for 120 s, where a 0.4 mA electrical foot-shock was subsequently applied to the mice for 2 s. After 60 s, another 0.4 mA electric shock was given to the mouse for 2 s. Following shocks, mice were left in the chamber for an additional 120 s. Twenty-four hours after training, each mouse was monitored in the same chamber for 5 min. Freezing time was automatically recorded and analyzed by the Freeze Detector System.

### Electrophysiology

Following behavior analysis, ex vivo hippocampal slices were prepared from WT and *Trem2* KO mice stereotaxically injected with either AAV-control or AAV-tau using methods described previously [31]. Briefly, mice were decapitated under deep terminal anesthesia, and brains were surgically removed in ice-cold, sucrose-based artificial cerebrospinal fluid (aCSF) (190 mM sucrose, 25 mM D-glucose; 25 mM NaHCO_3_, 3 mM KCl, 1.25 mM NaH_2_PO_4_, 5 mM MgSO_4_, 10 mM NaCl, and 0.5 mM CaCl_2_) saturated with carbogen (95% O_2_/5% CO_2_) at pH 7.4. A vibrating-blade microtome (Leica VT1000S) was used to cut 400-μm-thick coronal slices containing both cortex and hippocampus. Slices were transferred to a holding chamber containing a warmed (32 °C) aCSF formulation for recording (125 mM NaCl, 25 mM NaHCO_3_, 3.0 mM KCl, 1.25 mM NaH_2_PO_4_, 2.0 mM CaCl_2_, 1.0 mM MgSO_4_, and 10 mM D-glucose) saturated with carbogen (95% O_2_/5% CO_2_) at pH 7.4. Slices were left to recover at room temperature in oxygenated aCSF for at least 30 min before recording. Population spike amplitude was measured to determine synaptic transmission within the excitatory perforant pathway in acute hippocampal slices. Concentric bipolar stimulating electrodes were positioned in the middle molecular layer of the DG while recording electrodes were positioned in the dentate granule cell body layer. Stimuli (0.1 ms in duration) were applied at 0.05 Hz in increments of 20 μA from 0 to 200 μA, at each time-point, five recordings of evoked responses were averaged.

### Primary microglial and neuronal culture

Primary microglial cultures were prepared as described previously [3, 68]. Briefly, brains were removed from WT or *Trem2* KO mice at postnatal day 2–3. After removal of the meninges, brains were treated with a Papain Dissociation System (Worthington Biochemical Corporation) according to manufacturer’s specifications. Mixed glial cells were plated in flasks coated with poly-D-lysine and grown in DMEM containing 10% FBS (VWR Life Science Seradigm). Twenty-five nanograms per milliliter GM-CSF (R&D Systems) was added into the cultures after 5 days and removed before harvesting. Microglial cells were harvested twice by shaking (200 rpm, 60 min) 10–14 days after plating and subjected to various treatments within 24 h of harvest.

Neurons were dissected as described previously [58]. Primary neurons were dissected from embryonic days 17–18 (E17-E18) embryos from pregnant female C57BL/6 mice, and hippocampal and cortical neurons were isolated by microdissection from the cerebral cortex and hippocampus using a stereomicroscope. Tissue was dispersed by trypsin and DNase 1 digestion for 30 minutes at 37 °C, followed by trituration in DMEM+ penicillin /streptomycin + HEPES. Neurons were maintained separately on poly-D-lysine coated coverslips or seeded onto poly-D-lysine coated layers in microfluidic chamber systems. Neurons were cultured in Neurobasal Medium Plus supplemented with B27 Plus, glutamine, and penicillin/streptomycin, where half of the media was replaced every 2–3 days.

### 3-chamber interneuronal tau dispersion assay

A three-layer microfluidic chamber (TCND1000, Xona Microfluidics) was designed to reconstitute intraneuronal tau dispersion through cells within an intermediary layer, with microgrooves adjoining the three chambers. Two side-reservoirs connected the poly-D-lysine coated culture chambers to facilitate neuronal adhesion. Primary cortical neurons from wildtype C57BL/6 J E18 embryos were dissected as previously described [58] (see “Primary microglial and neuronal culture”) and 2 × 10^5^ cells were plated in chamber layers 1 and 3 in 50% neurobasal with B27 medium and 50% DMEM/F12 with FBS medium, and allowed to adhere overnight. The following day, medium was changed to complete neurobasal with B27 and maintained at 37 °C in 5% CO_2_. Neurons in layer 1 were transduced with 1 × 10^10^ VP/ml AAV-tau particles at DIV2. At DIV7, primary WT or *Trem2* KO microglia were detached from mixed glial cultures and 1 × 10^4^ cells were seeded into chamber layer 2 in 50% Neurobasal with B27 containing/50% DMEM with 10% FBS, 25 ng/ml GM-CSF (a control without microglia in layer 2 was also included); and neurons/microglia were cultured for another 7 days. At DIV14, chambers were fixed and stained to visualize tubulin (#5568, Cell Signaling Technology), Iba1 (ab5076, Abcam) and human tau (T13, Biolegend) by fluorescence microscopy.

Intensity of human tau (T13) staining and neuronal density in layers 1 and 3 were calculated from intensity measurements using Imaris (Bitplane) in imaged fields, and neuronal number were quantified by DAPI measurements in layers 1 and 3 (normalized per cm^2^). Iba1-positive microglia were also quantified in chamber layer 2, and overlap in human tau with Iba-1 positive microglia were quantified, and normalized to Iba1/tau-positive microglia in WT layer 2 (set to 1.0).

### Microglia tau uptake

Binding/uptake of tau oligomers in cultured WT and *Trem2* KO microglia was measured using purified recombinant 2N4R human tau 1–441 (500 μg/ml, #AS-55556-50, AnaSpec) oligomerized in 30 μM heparin (#07980, StemCell Technologies Inc.) for 24 h. Tau oligomers were subsequently conjugated to Alex555 (“tau-555”) using an Alexa Fluor 555 Microscale Protein Labeling Kit (#A30007, ThermoFisher) according to the manufacturer’s instructions. Binding/uptake of tau-Alexa Fluor 555 was assayed in microglia cultures seeded at 50,000 cells in 24-well plates, and tau-555 binding/uptake was measured in real time at a final concentration of 10 μg/ml where a fixed area in each well was serially imaged every 15 min using a Nikon N-SIM microscope. Serial confocal images were acquired for 10 h, and tau-555 intensity/area was quantified. Fluorescence intensity was normalized to microglia cell number using automated IMARIS imaging software (Bitplane); fluorescence thresholds for tau-555 were set to a value of 10, and individual cells were identified by differential interference contrast (DIC) imaging. Fluorescence measurements normalized to cell number from 9 total confocal images from 3 independent microglia batches/experiments (three independent wells per batch) at time points ranging from 15 to 180 min (for all experiments). The phagocytic index (PI) for varying time points was then calculated using the following formula: PI = I_t_ / I_15_, where I_t_ represents averaged fluorescence intensity at various time points and I_15_ represents fluorescence intensity at 15 min following addition of tau-555. The resulting value represents fold change of tau-555over the 15 min time point for each microglia culture. Ratios were then normalized to the PI value in WT microglia under control conditions at 15 min (where WT microglia under control conditions/15 min are set to 1.0) for each experiment.

### Flow cytometry and TUNEL analysis

#### Flow cytometry for tau oligomer uptake

Tau oligomers were conjugated to an Alexa-488 fluor label using an Alexa Fluor™ 488 Protein Labeling Kit (A10235, ThermoFisher); WT and *Trem2* KO microglia were incubated with tau-Alexa488 (tau488) for 6 hours. Microglia were washed in 1xPBS, harvested and fixed with 4% PFA. Fluorescent signals were detected and quantified by flow cytometry (Novocyte, ACEA Bioscience). WT microglia (“cell only”) without tau uptake was used as an unlabeled control.

#### TUNEL analysis

Effects of LPS and ATP treatment on cell death in WT and *Trem2* KO microglia were quantified by TUNEL labeling and flow cytometry analysis. 1 × 10^6^ WT and *Trem2* KO microglia left untreated or treated with 1 μg/ml LPS (#L3024, Sigma-Aldrich) for 3 h, and 5 mM ATP, or WT microglia exposed to 10 μM camptothecin for 3 h were processed for TUNEL staining using the Apo-Direct TUNEL Assay Kit (APT110, Millipore Sigma) according to the manufacturer’s recommendations, and subjected to flow cytometry and FlowJo analysis. TUNEL staining in WT and *Trem2* KO microglia under varying treatments was performed using the Click-IT Plus TUNEL assay system (C10617, ThermoFisher), and stained cells were imaged by confocal microscopy.

### Induction and purification of microglia exosomes

Exosomes were purified from primary microglia cultures as described previously [2, 54]. Briefly, 1 × 10^7^ WT and *Trem2* KO microglia cultured under untreated conditions or incubated with 2.5 μg/ml tau oligomers for 24 h; cells were then washed in 1xPBS and incubated in DMEM with 10% exosome-depleted FBS (A2720803, ThermoFisher) and treated with 1 μg/ml LPS (#L3024, Sigma-Aldrich) for 3 h, and 5 mM ATP for 15 mins. Conditioned media (5 ml) was collected and centrifuged at 2000×g, and supernatant subsequently centrifuged at 10,000×g for 30 min at 4 °C to remove cell debris. Supernatants were then diluted to 10 ml in 1x PBS and centrifuged at 100,000×g for 90 mins. To precipitate exosomes, pellets were then resuspended in 10 ml 1xPBS and re-centrifuged at 100,000×g to remove contaminants and non-exosomal debris. Resulting pellets were then resuspended in 50 μl 1xPBS for electron microscopy (EM), ELISA, or RIPA buffer (50 mM Tris-HCl pH 7.4, 150 mM NaCl, 1% NP-40, 0.1% SDS, 0.5% DOC) for immunoblot analysis.

### Electron microscopy

Formvar-carbon-coated copper grids (100 mesh, Electron Microscopy Sciences, Hatfield, PA) were placed on 20 μl drops of each sample solution displayed on a Parafilm sheet. After allowing material to adhere to the grids for 10 minutes, grids were washed 3 times by rinsing through 200 μl drops of milli-Q water before being left for 1 min on 2% (wt/vol) uranyl acetate (Ladd Research Industries, Williston, VT). Excess solution was removed with Whatman 3MM blotting paper, and grids were left to dry for a few minutes before viewing. Grids were examined using a JEOL JEM-1400Plus transmission electron microscope operating at 80 kV. Images were recorded using a Gatan OneView 4 K digital camera.

### Immunoblot and ELISA analysis

Exosome preparations or lysates from primary microglia were generated/resuspended in RIPA buffer in the presence of protease and phosphatase inhibitors (#78430, ThermoFisher). Proteins were separated by SDS-PAGE using 4–20% gradient gels, transferred onto nitrocellulose, and blocked in 5% non-fat milk in 1xPBS. Blots were then probed with primary antibodies overnight in 5% BSA/1xPBS, washed in 1xPBS with 0.1% tween-20, and probed with HRP-conjugated secondary antibodies. Blots were then incubated with ECL and immunoblot signals were acquired using a Chemidoc imaging system (BioRad). ELISA measurements for exosomal human tau were performed using ELISA kits for tau (KHB0041, ThermoFisher) according to specifications supplied by the manufacturer. Primary antibodies used for immunoblot analysis include Alix (1:1000, 2171 Cell Signaling), T13 (htau) and actin (1:5000, A5441 Sigma).

### Sample preparation for proteomics analysis and LC-MS/MS

Primary *Trem2* KO or WT microglia were left untreated, or treated with tau oligomers (2.5 μg/ml for 24 h), or 1 μg/ml LPS for 3 h, and 5 mM ATP for 15 mins (run 2), and cell pellets were lysed in UAB buffer (8 M urea, 50 mM ammonium bicarbonate (ABC) and Benzonase 24 U/100 ml). Protein concentration was determined using BCA assays (ThermoFisher) according to the manufacturer’s instructions. Proteins were then reduced by the addition of 5 mM tris(2-carboxyethyl) phosphine (TCEP) at 30 °C for 60 min, followed by alkylation of cysteines with 15 mM iodoacetamide (IAA) for 30 minutes in the dark at room temperature. Urea concentration was reduced to 1 M by adding 50 mM ammonium bicarbonate. Samples were digested overnight with Lys-C/trypsin (Promega) at room temperature with constant agitation at a 1:25 enzyme:protein ratio. Following digestion, samples were acidified using 0.1% FA and desalted using AssayMap C18 cartridges mounted on an Agilent AssayMap BRAVO liquid handling system. Cartridges were sequentially conditioned with 100% acetonitrile (ACN) and 0.1% FA; samples were then loaded, washed with 0.1% FA, and eluted with 60% ACN, 0.1% FA. Peptide concentration was determined using a NanoDrop spectrophotometer (Thermo Fisher).

Samples were subjected to mass spectrometry analysis using an EASY nanoLC system (ThermoFisher). Buffer A consisted of H_2_O/0.1% FA; Buffer B consisted of 80% ACN/0.1% FA. Samples were separated over a 90-min gradient of increasing Buffer B on analytical C18 Aurora column (75 μm × 250 mm, 1.6 μm particles; IonOpticks) at a flow rate of 300 nL/min. The mass spectrometer was operated in positive data-dependent acquisition mode, and the Thermo FAIMS Pro device was set to standard resolution with the temperature of FAIMS inner and outer electrodes set to 100 °C. A three-experiment method was set up where each experiment utilized a different FAIMS Pro compensation voltage: - 50, − 70, and − 80 Volts, and each of the three experiments had a 1 second cycle time. A high resolution MS1 scan in the Orbitrap (m/z range 350 to 1500, 60 k resolution at *m/z* 200, AGC 4e5 with maximum injection time of 50 ms, RF lens 30%) was collected in top speed mode with 1-second cycles for the survey and the MS/MS scans. For MS2 spectra, ions with charge state between + 2 and + 7 were isolated with the quadrupole mass filter using a 0.7 m/z isolation window, fragmented with higher-energy collisional dissociation (HCD) with normalized collision energy of 30% and the resulting fragments were detected in the ion trap as rapid scan mode with AGC of 5e4 and maximum injection time of 35 ms. The dynamic exclusion was set to 20 sec with a 10 ppm mass tolerance around the precursor.

### Proteomic data analysis

Raw files were searched with SpectroMine software (Biognosys, version 2.7.210226.47784) using the BGS default settings. The search criteria were set as follows: full tryptic specificity was, 2 missed cleavages were allowed, carbamidomethylation (C) was set as fixed modification and oxidation (M) as a variable modification. The false identification rate was set to 1%. Spectra were searched against the curated Uniprot *mus musculus* database including common contaminants from the GPM cRAP sequences. Data was further processed using the MSstats package (version 4.2) in R [14]. We avoided use of imputation of missing values prior to statistical test using MSstats; instead we calculated a pseudo Log_2_ fold-change (L2FC), adj.pvalue and pvalue of proteins completely missing in one condition after failing to perform the statistical test. The imputed (pseudo) L2FC was calculated as the sum of intensities of the protein (i.e., sum of feature intensities of a given protein within a given sample) across all replicates of the same group that it was detected, divided by 3.3. On the other hand, the imputed pvalue and adj.pvalue was calculated by dividing 0.05 or 0.1, respectively, by the number of replicates that a given protein was confidently identified multiplied by the number of features quantified. Therefore, the imputed L2FC gives an estimate of the protein abundance in condition that it is detected, while the imputed pvalue or adj.pvalue reports the confidence of the imputation in the sense of consistency of protein detection in the group that it is detected.

Proteins with an adjusted *p* value< 0.05 (adjp< 0.05) were selected as significantly differentially expressed proteins (DEPs). GO analysis was performed by entering DEPs into the GO DAVID input interface, and KEGG, Biological Process (BP), Cellular Component (CC) and Molecular Function (MF) categories were retrieved for each query [29, 30]. Principal Component Analysis (PCA) was carried out in R version 4.1.2 with PCATools package (version 2.6.0) using log2 protein intensity for all proteins summarized by *dataProcess* function from MSstats (version 4.2). Contaminant proteins (non-murine, “*Bos taurus*” proteins) or proteins with gene names that failed to map from gene ID’s were manually removed from the datasets prior to analysis. To calculate z-score values within each replicate, row (protein-wise) z-scores were computed in R version 4.0.2 using the scale function by subtracting mean intensity of each protein from the corresponding intensities of the biological replicates, and dividing the resulting values by the standard deviation of the intensities.

### Tau internalization and quantification

WT and *Trem2* KO microglia were seeded at a density of 0.2 million cells on coverslips on 24-well plates, and treated with 2.5 μg/ml tau oligomers for 0.5, 2, 4 and 24 h. Cells were then fixed and stained with antibodies to visualize colocalization of recombinant human tau (T13) with Rab5, Rab7, LAMP1, CD63 and Tsg101. Images were acquired by confocal microscopy and overlapping signals from tau and intracellular markers were quantified; manual identification, demarcation and quantification of regions of overlap were performed using Imaris, and normalized to the total area of tau staining in fluorescence images.

### In vitro exosomal tau seeding assay

Tau seeding was performed using a FRET biosensor HEK293T cell line stably expressing tau-RD P301S-CFP and P301S-YFP (tau-RD cells) as described previously [26]. To assay tau seeding capacity of exosomes purified from cultured microglia, exosomes were purified from WT or *Trem2* KO microglia pre-incubated with tau oligomers and induced with LPS/ATP and resuspended in 50 μl PBS. Tau-RD cells were then exposed to 20 μl purified exosomes and transduced with Lipofectamine 2000 (#11668019, ThermoFisher) for 48 hours, and FRET activity from tau aggregation was visualized by excitation at 405 nm and consequent fluorescence at 525/50 nm was imaged using a Zeiss LSM 710 microscope. DIC images were concurrently acquired in FRET images and used to normalize FRET activity according to cell number. FRET area was calculated from positive cell signals in tau-RD cells through defined FRET-positive regions in Imaris, and FRET-positive area (μm^2^) was normalized over cell number; cells without FRET signals were included in the analyses.

### Stereotaxic exosome injection and analysis in vivo

Exosomes were prepared from 3 × 10^7^ tau-loaded and untreated (control), LPS/ATP-treated WT and *Trem2* KO microglia as described under “Induction and purification of microglia exosomes”. Exosome pellets were resuspended in 20 μl sterile 1xPBS and 2 μl of the exosome preparations purified from WT and *Trem2* KO microglia were stereotaxically injected into the DG region (coordinates: AP, − 2.0 mm; ML, ± 1.3 mm; DV, 2.1 mm) in 6 month C57BL/6 WT mice. Three weeks post-injection, mice were perfused with 4% PFA and stained with AT8 (MN1020, Thermo Fisher), β3-Tubulin (5568, Cell Signaling Technology) and DAPI to examine tau pathology. AT8-positive cells were scored in 2.5 × 10^5^ μm^2^ imaging areas in independently-injected animals.

### Statistical analysis

All statistical analyses were performed using R scripts (proteomics) or Graphpad Prism as indicated.

## Supplementary Information

**Additional file 1: Fig. S1.** (A) AAV particles expressing human tau were stereotactically injected into the medial entorhinal cortex (MEC) of wildtype (WT) or *Trem2* KO mice at 4 months of age, and histological images from the MEC (upper panels) or DG (lower panels) were obtained by confocal microscopy 5 weeks post-injection. Representative images from the MEC were stained with AT8 (pS202, T205 tau) and AT180 (pT231 tau) antibodies (green), in addition to Iba1 (purple), and DAPI (blue) as indicated. Bar, 100 μm. (B) Wildtype (WT) mice were injected with AAV-GFP and AAV-tau in combination (1 × 10^9^ AAV particles each), and GFP (green), AT8 (pS202, T305 tau, red) and DAPI (nuclei, blue) staining within the MEC and hippocampal dentate gyrus (DG) was imaged by confocal microscopy 5 weeks following injection. Bar = 50 μm. (C) Magnified images of MEC and DG in WT and *Trem2* KO animals 5 weeks following AAV-tau MEC injection. Histological slices were stained for AT8 (re-colored in purple), Cd68 (re-colored green), and P2y12 (red) and DAPI (nuclei, blue). Bar = 50 μm.

**Additional file 2: Fig. S2.** *Trem2* deletion affects tau endocytic trafficking without affecting microglial tau uptake. (A) WT or *Trem2* KO microglia were incubated with tau 555 oligomers, and uptake was measured by fluorescence real-time confocal imaging. Images selected at 30 minute intervals are shown from 30′ to 4 h from the 15′ starting point. (B) Relative tau phagocytic uptake for 6 h was quantified from 3 independent cultures (mean ± SE). Phagocytic index was determined by measuring average fluorescence intensity in individual microglia WT or *Trem2* KO cultures in comparison to the 1 h timepoint, and normalized to WT at the 1 h timepoint (set to 1.0). (C) Quantification of tau uptake by FACS analysis. WT (blue) or *Trem2* KO (red) microglia were incubated with tau-488 oligomers for 6 h and tau-positive cells were quantified. WT microglia without tau (green) were included as a control. (D-F) Effects of *Trem2* deletion on tau uptake into endosomal and lysosmal compartments. WT or *Trem2* KO microglia were treated with 2.5 μg tau oligomers for the time indicated, and stained for human tau (T13, green) and Rab5 (D), Rab7 (E), and LAMP1 (F) (red) and nuclei (DAPI, blue). Percentage area of tau within red intracellular is calculated from 3 experiments during the tau timecourse is shown in the adjacent graphs (mean ± SE). Bar = 5 μm. Graphs represent mean ± SE.

**Additional file 3: Fig. S3.** TUNEL analysis of WT and *Trem2* KO microglia with LPS/ATP treatment. (A) WT and *Trem2* KO microglia untreated or treated with 1μg/ml LPS for 3 h and 5 mM ATP for 15 mins, or WT microglia treated with 10uM camptothecin for 3 h were stained for TUNEL, and TUNEL positive cells were quantified by flow cytometry analysis. A representative gate of untreated WT microglia is shown. (B) Cell population overlays of untreated (WT control, red), or camptothecin-treated (WT with camptothecin, blue), WT untreated (brown) and LPS/ATP treated (orange), or *Trem2* KO untreated (dark green) and LPS/ATP-treated (light green) TUNEL-labeled peaks as detected by flow cytometry analysis. (C) Representative fluorescence images of WT and *Trem2* KO microglia untreated or treated with camptothecin, or LPS/ATP as indicated. TUNEL staining was performed using the Click-IT in situ labeling system shown in green (TUNEL) merged with nuclei staining (DAPI) in blue. Bar = 20 μm.

**Additional file 4: Fig. S4.** Proteomic analysis of lysates from *Trem2* KO and WT microglia. (A, B) Volcano plots depicting differentially-enriched (expressed) proteins in lysates from *Trem2* KO and WT microglia untreated or induced with tau oligomers (run 1) (A) or LPS/ATP run 2 (B). Differentially expressed proteins (DEPs) are derived from *Trem2* KO vs WT comparisons under steady-state (untreated) or with tau or LPS/ATP; and tau or LPS/ATP vs untreated comparisons in *Trem2* KO or WT microglia lysates as indicated. Significant proteins were defined by an adjusted *p*-value of less than 0.05, and samples with proteins absent under one condition (e.g. Trem2) are marked by “X’s”. *Trem2* was identified in WT lysates, and was absent in KO lysates as indicated in purple (KO vs WT, untreated). (C) 64 overlapping DEPs in *Trem2* KO (“KO”) microglia in runs 1 and 2; heatmaps indicate Log_2_ fold-change (FC) and adj *p*-values. Identification of Trem2 (downregulated in KO) is indicated by the black arrow. Condensed z-scores for 6 replicates from run 1 (left) and run 2 (right) are shown on the adjacent heatmaps to the right (WT and KO), “NA” values derived from missing peptide values are indicated in gray. (D) Overlapping DEPs identified in runs 1 (left) and 2 (right Venn diagrams). In run 1, 385 DEPs in KO vs WT were unchanged with tau (top), and 884 tau-responsive DEPs were observed in both WT and KO microglia. In run 2, 142 DEPs in KO vs WT were unchanged with LPS/ATP (top), and 1373 LPS/ATP-responsive DEPs were observed in both WT and KO microglia.

**Additional file 5: Fig. S5.** Proteomic analysis of lysates from *Trem2* KO and WT microglia (continued). (A) Effects of *Trem2* deletion on DAM-responsive proteins with tau or LPS/ATP treatment. Log_2_ fold-change of DAM-associated DEPs identified in pairwise comparisons from run 1 (tau) or run 2 (LPS/ATP, gray) and adj p-values are indicated in the left heatmaps. Z-score heatmaps depict calculated z-scores of 6 replicates from run 1 (left) and run 2 (right) are shown on the adjacent heatmaps to the right; “NA” values derived from missing peptide values are indicated in gray. (B,C) Overlapping DEPs in response to both tau and LPS/ATP in WT (B) and *Trem2* KO (C) microglia are depicted in the Venn diagrams, and Log2 fold-change, adj p-values and z-scores of the 302 WT (B) or 473 KO (C) DEPs are plotted in the heatmaps indicated. Overlapping WT (B) and KO (C) DEPs were also plotted for Log_2_ fold-change distribution in the bottom scatterplots for change in response to tau (x-axis) and LPS/ATP (y-axis).

**Additional file 6: Fig. S6.** Effects of *Trem2* deletion on proteomic changes with tau and LPS/ATP treatment (continued). (A) Gene Ontology (GO DAVID) analysis of 141 overlapping genes that change in response to tau and LPS/ATP in both WT and KO microglia. Number of DEPs and FDR for the top 15 GO BP (left graphs) and KEGG categories (right graphs) are shown. NFkB categories in BP and KEGG analysis are highlighted in purple. (B) Overlap in DEPs identified in WT (gray) and *Trem2* KO (purple) response to tau oligomers (proteomics, this study), and DEGs identified in WT microglia treated with tau fibrils [62] (violet) by RNAseq analysis. (C) Z-scores for 6 replicates from run 1 (left) and run 2 (right) are shown on the left, “NA” values derived from missing peptide values are indicated in gray. Adjacent heatmaps depicting Log_2_ fold-change and adj p-values of 52 DEPs identified by GO KEGG analysis; Log_2_ fold-change heatmaps for “tau-responsive” NFkB DEPs (from run 1) are also shown, Nfkb1 and 2 are indicated by black arrows. (D) Heatmaps depicting Log_2_ fold-change and adj p-values of potential exosome-associated DEPs in the mouse microglia Exocarta database. Z-scores for 6 replicates from run 1 (left) and run 2 (right) are shown on the adjacent heatmaps to the right, “NA” values derived from missing peptide values are indicated in gray.

**Additional file 7: Fig. S7.** Effects of *Trem2* deletion on proteomic changes with tau and LPS/ATP treatment (continued). (A) Heatmaps depicting Log_2_ fold-change and adj p-values of SNX and VPS DEPs by pairwise comparison of the treatment groups indicated in runs 1 and 2. Z-scores for 6 replicates from run 1 (left) and run 2 (right) are shown on the adjacent heatmaps to the right, “NA” values derived from missing peptide values are indicated in gray. (B) Heatmaps depicting Log_2_ fold-change and adj p-values of GO MF “tau protein binding” DEPs by pairwise comparison of the treatment groups indicated in runs 1 and 2. Z-scores for 6 replicates from run 1 (left) and run 2 (right) are shown on the adjacent heatmaps to the right, “NA” values derived from missing peptide values are indicated in gray. (C) Representative images of tau loading (2.5 μg/ml, 24 h) in WT and *Trem2* KO microglia with and without tau and GW4869 (inhib). Cells were fixed and stained to visualize Iba1 (purple), tau (green) and nuclei (DAPI, blue) as indicated. Bar = 10 μm.

**Additional file 8: Table S1.** Proteomic analysis of cell lysates from *Trem2* KO and WT microglia under steady-state and tau (run 1) and LPS/ATP-treated (run 2) conditions; includes raw data, Log_2_ fold-change and adj-*p* values between various treatment groups, and z-score values of DEPs.

**Additional file 9: Table S2.** DEP analysis from *Trem2* KO and WT microglia under steady-state and tau (run 1) or LPS/ATP-treated (run 2) conditions; GO KEGG, BP (Biological Process), CC (Cellular Component), and MF (Molecular Function) analysis using GO DAVID.

## Data Availability

All data generated in this study are included in this published article. The proteomics dataset supporting the conclusions of this article is freely available on the MassIVE database, and can be accessed at ftp://massive.ucsd.edu/MSV000090168/ and ftp://massive.ucsd.edu/MSV000089092. Additional datasets supporting the conclusions of this article are included within the article (Figs. 1, 2, 3, 4, 5, 6 and 7) and its additional files (Figs. S1, S2, S3, S4, S5, S6 and S7, Tables S1 and S2).

## Abbreviations

AD: Alzheimer’s disease
DAM: Disease-associated microglia
TREM2: Triggering receptor expressed on myeloid cells 2
WT: Wildtype
FRET: Fluorescence resonance energy transfer
MEC: Medial entorhinal cortex
DG: Dentate gyrus
GWAS: Genome-wide association studies
AAV: Adeno-associated virus
MVB: Multivesicular body
LPS: Lipopolysaccharide
ATP: Adenosine tri-phosphate
GO: Gene Ontology
DEP: Differentially-expressed protein
CC: Cellular component
BP: Biological process
KEGG: Kyoto encyclopedia of Genes and Genomes
ELISA: Enzyme-linked immunoassay
iPSC: Induced pluripotent stem cells
MGnD: Neurodegenerative microglia
FACS: Fluorescence-activated cell sorting
EM: Electron microscopy
