# Supplementary figures and images for: Trem2 deletion enhances tau dispersion and pathology through microglia exosomes
Source: Mol Neurodegener. 2022 Sep 2;17:58. doi: 10.1186/s13024-022-00562-8 (PMC9438095; doi:10.1186/s13024-022-00562-8)

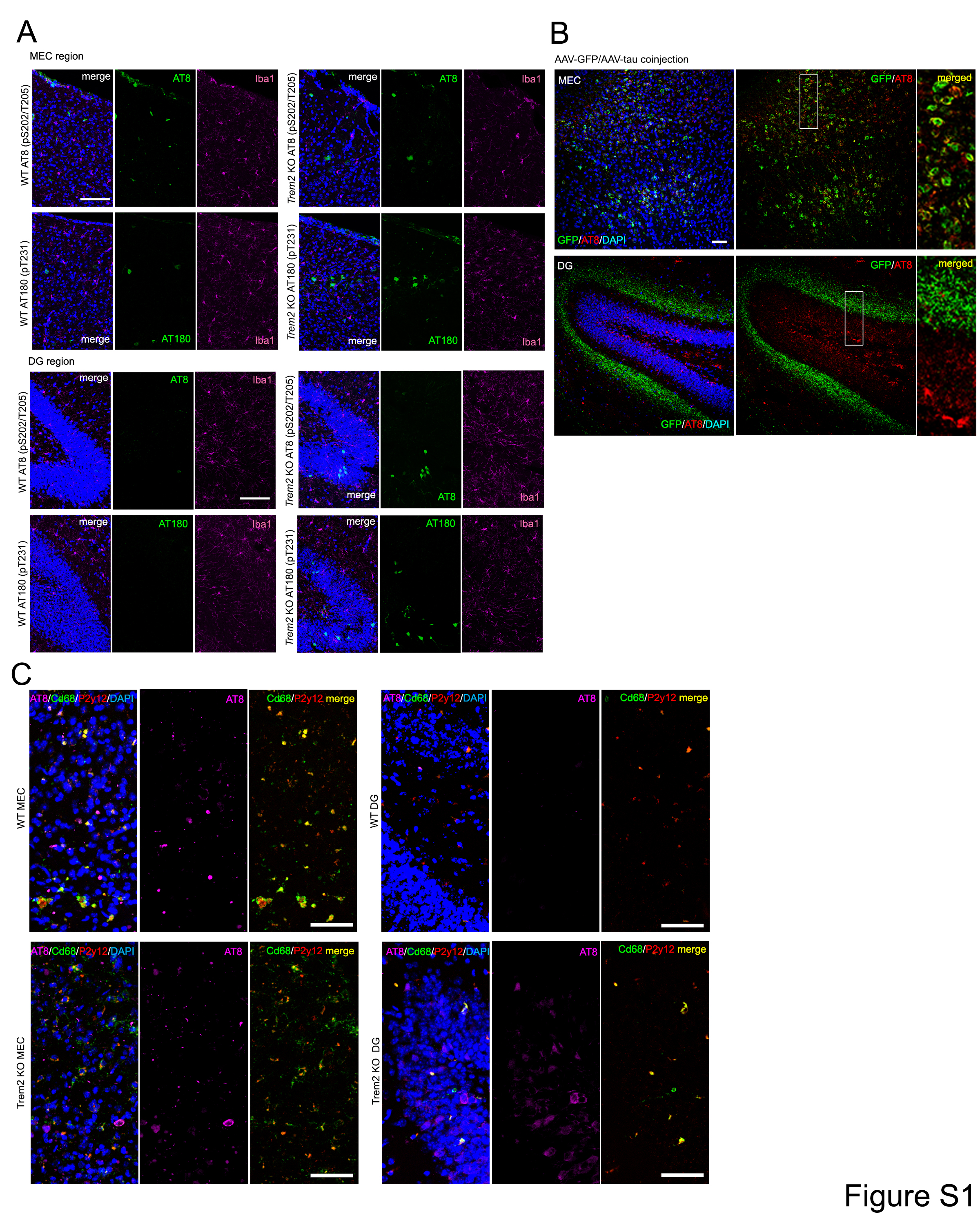

Supplement: Supplementary file 1 — Additional file 1: Fig. S1. (A) AAV particles expressing human tau were stereotactically injected into the medial entorhinal cortex (MEC) of wildtype (WT) or Trem2 KO mice at 4 months of age, and histological images from the MEC (upper panels) or DG (lower panels) were obtained by confocal microscopy 5 weeks post-injection. Representative images from the MEC were stained with AT8 (pS202, T205 tau) and AT180 (pT231 tau) antibodies (green), in addition to Iba1 (purple), and DAPI (blue) as indicated. Bar, 100 μm. (B) Wildtype (WT) mice were injected with AAV-GFP and AAV-tau in combination (1 × 10^9^ AAV particles each), and GFP (green), AT8 (pS202, T305 tau, red) and DAPI (nuclei, blue) staining within the MEC and hippocampal dentate gyrus (DG) was imaged by confocal microscopy 5 weeks following injection. Bar = 50 μm. (C) Magnified images of MEC and DG in WT and Trem2 KO animals 5 weeks following AAV-tau MEC injection. Histological slices were stained for AT8 (re-colored in purple), Cd68 (re-colored green), and P2y12 (red) and DAPI (nuclei, blue). Bar = 50 μm. [file 13024_2022_562_MOESM1_ESM.jpg]

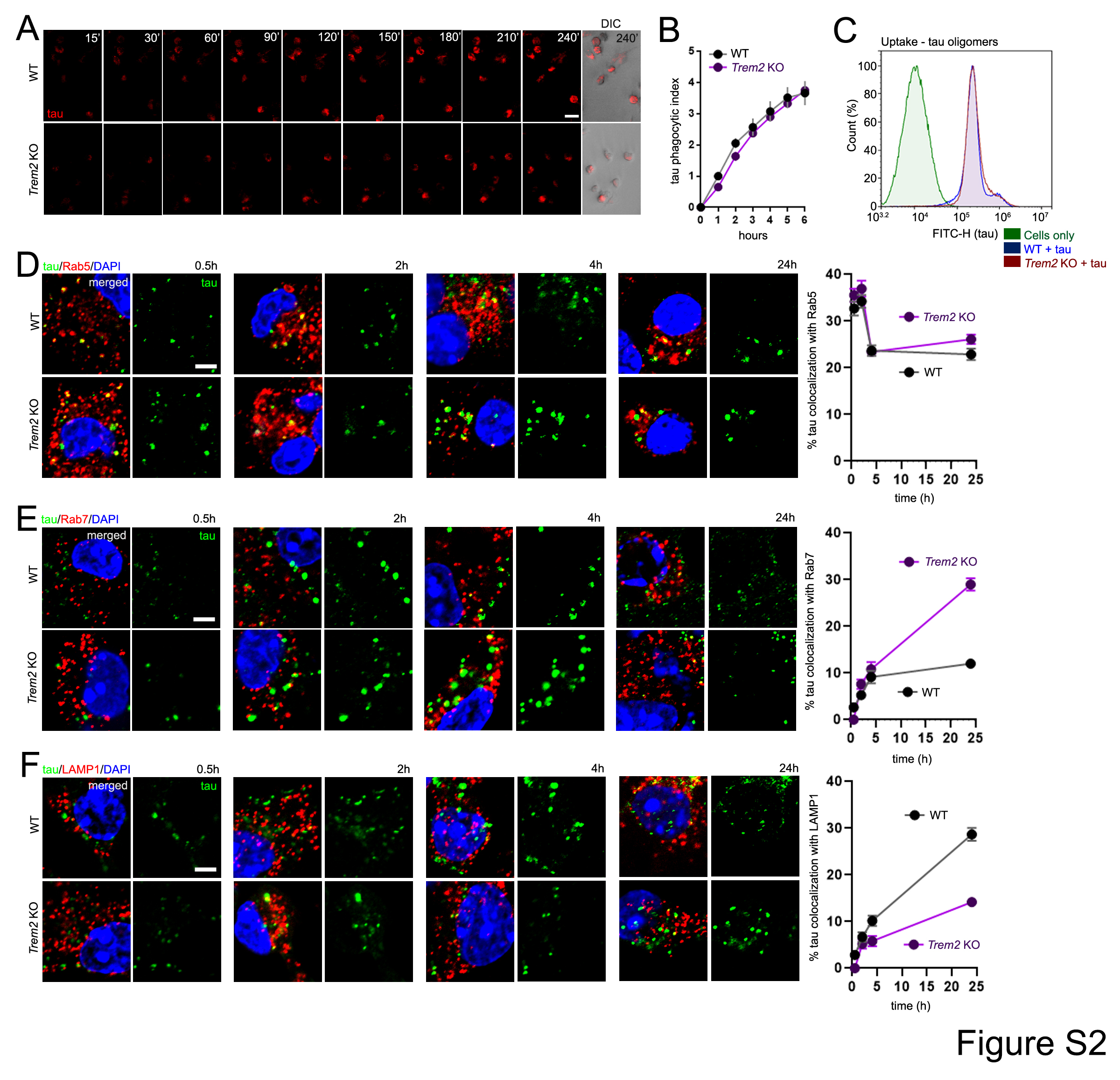

Supplement: Supplementary file 2 — Additional file 2: Fig. S2. Trem2 deletion affects tau endocytic trafficking without affecting microglial tau uptake. (A) WT or Trem2 KO microglia were incubated with tau 555 oligomers, and uptake was measured by fluorescence real-time confocal imaging. Images selected at 30 minute intervals are shown from 30′ to 4 h from the 15′ starting point. (B) Relative tau phagocytic uptake for 6 h was quantified from 3 independent cultures (mean ± SE). Phagocytic index was determined by measuring average fluorescence intensity in individual microglia WT or Trem2 KO cultures in comparison to the 1 h timepoint, and normalized to WT at the 1 h timepoint (set to 1.0). (C) Quantification of tau uptake by FACS analysis. WT (blue) or Trem2 KO (red) microglia were incubated with tau-488 oligomers for 6 h and tau-positive cells were quantified. WT microglia without tau (green) were included as a control. (D-F) Effects of Trem2 deletion on tau uptake into endosomal and lysosmal compartments. WT or Trem2 KO microglia were treated with 2.5 μg tau oligomers for the time indicated, and stained for human tau (T13, green) and Rab5 (D), Rab7 (E), and LAMP1 (F) (red) and nuclei (DAPI, blue). Percentage area of tau within red intracellular is calculated from 3 experiments during the tau timecourse is shown in the adjacent graphs (mean ± SE). Bar = 5 μm. Graphs represent mean ± SE. [file 13024_2022_562_MOESM2_ESM.jpg]

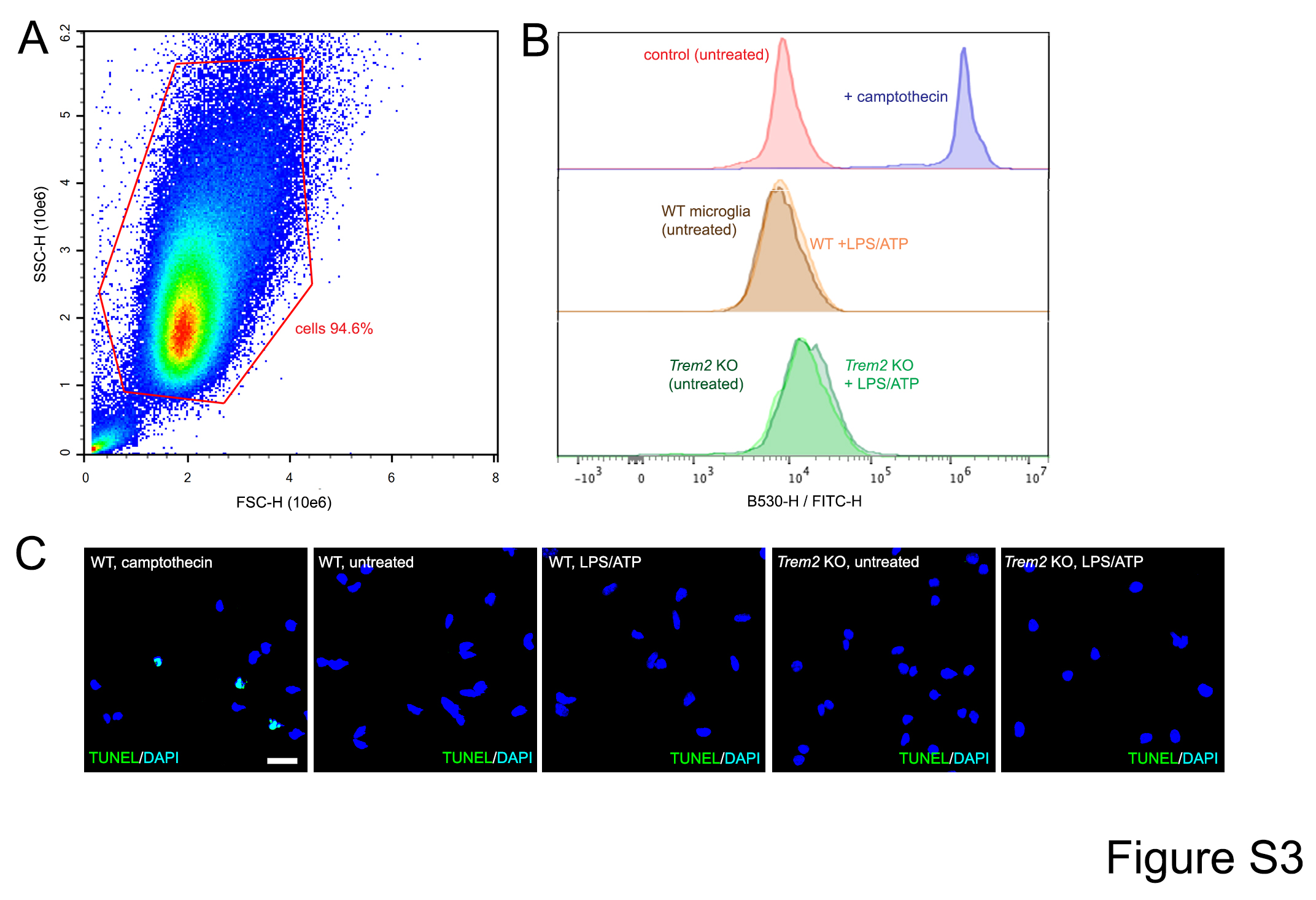

Supplement: Supplementary file 3 — Additional file 3: Fig. S3. TUNEL analysis of WT and Trem2 KO microglia with LPS/ATP treatment. (A) WT and Trem2 KO microglia untreated or treated with 1μg/ml LPS for 3 h and 5 mM ATP for 15 mins, or WT microglia treated with 10uM camptothecin for 3 h were stained for TUNEL, and TUNEL positive cells were quantified by flow cytometry analysis. A representative gate of untreated WT microglia is shown. (B) Cell population overlays of untreated (WT control, red), or camptothecin-treated (WT with camptothecin, blue), WT untreated (brown) and LPS/ATP treated (orange), or Trem2 KO untreated (dark green) and LPS/ATP-treated (light green) TUNEL-labeled peaks as detected by flow cytometry analysis. (C) Representative fluorescence images of WT and Trem2 KO microglia untreated or treated with camptothecin, or LPS/ATP as indicated. TUNEL staining was performed using the Click-IT in situ labeling system shown in green (TUNEL) merged with nuclei staining (DAPI) in blue. Bar = 20 μm. [file 13024_2022_562_MOESM3_ESM.jpg]

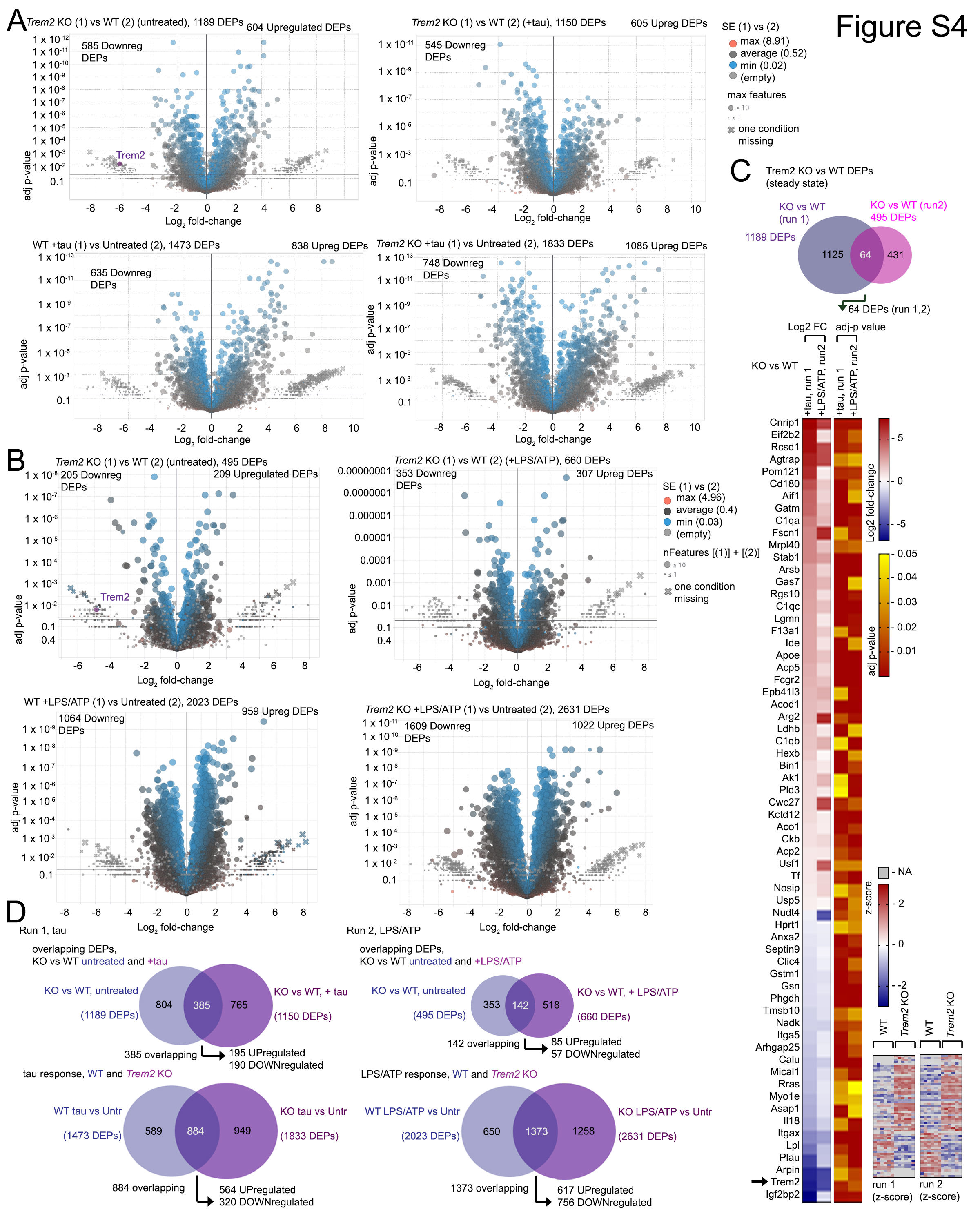

Supplement: Supplementary file 4 — Additional file 4: Fig. S4. Proteomic analysis of lysates from Trem2 KO and WT microglia. (A, B) Volcano plots depicting differentially-enriched (expressed) proteins in lysates from Trem2 KO and WT microglia untreated or induced with tau oligomers (run 1) (A) or LPS/ATP run 2 (B). Differentially expressed proteins (DEPs) are derived from Trem2 KO vs WT comparisons under steady-state (untreated) or with tau or LPS/ATP; and tau or LPS/ATP vs untreated comparisons in Trem2 KO or WT microglia lysates as indicated. Significant proteins were defined by an adjusted p-value of less than 0.05, and samples with proteins absent under one condition (e.g. Trem2) are marked by “X’s”. Trem2 was identified in WT lysates, and was absent in KO lysates as indicated in purple (KO vs WT, untreated). (C) 64 overlapping DEPs in Trem2 KO (“KO”) microglia in runs 1 and 2; heatmaps indicate Log2 fold-change (FC) and adj p-values. Identification of Trem2 (downregulated in KO) is indicated by the black arrow. Condensed z-scores for 6 replicates from run 1 (left) and run 2 (right) are shown on the adjacent heatmaps to the right (WT and KO), “NA” values derived from missing peptide values are indicated in gray. (D) Overlapping DEPs identified in runs 1 (left) and 2 (right Venn diagrams). In run 1, 385 DEPs in KO vs WT were unchanged with tau (top), and 884 tau-responsive DEPs were observed in both WT and KO microglia. In run 2, 142 DEPs in KO vs WT were unchanged with LPS/ATP (top), and 1373 LPS/ATP-responsive DEPs were observed in both WT and KO microglia. [file 13024_2022_562_MOESM4_ESM.jpg]

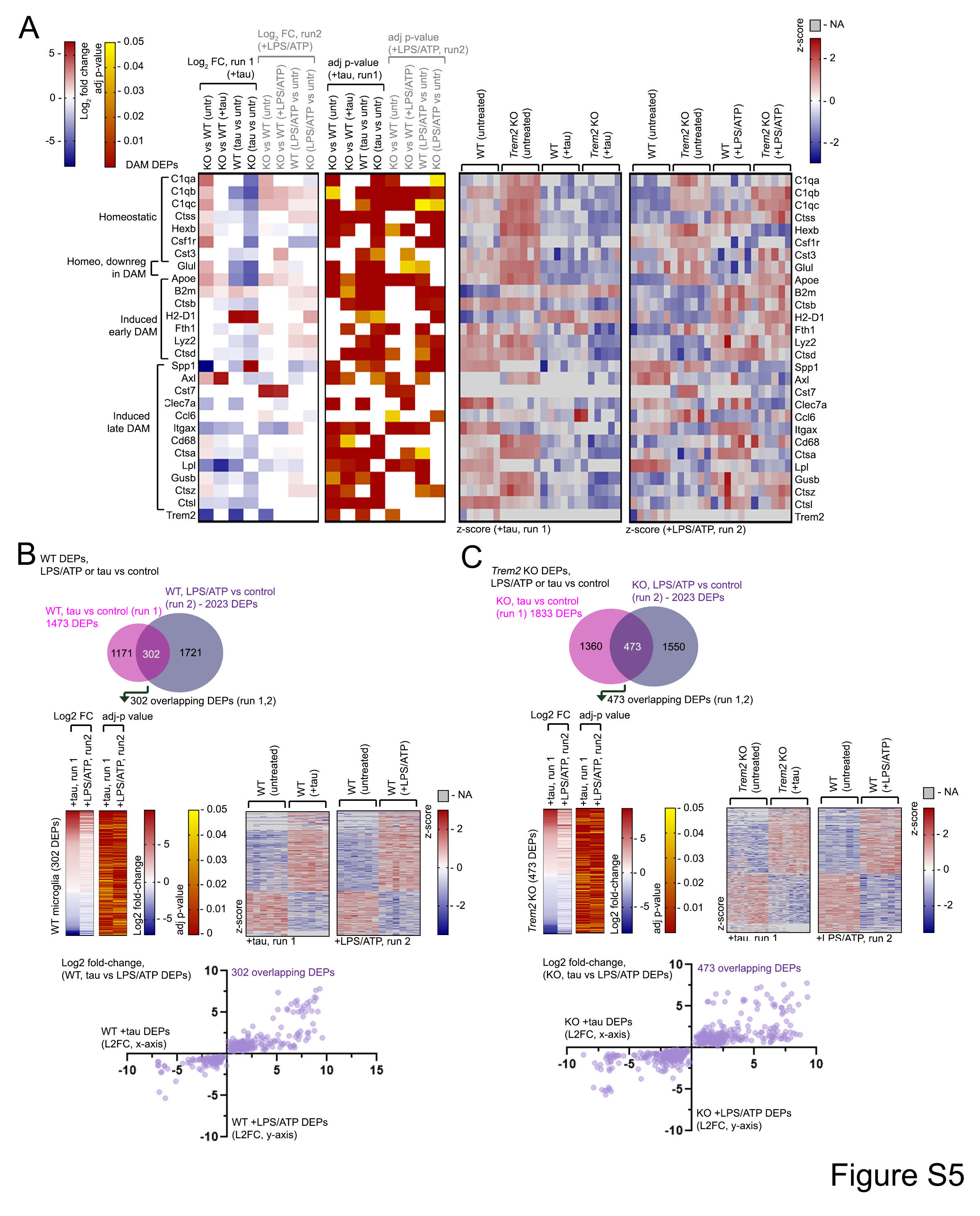

Supplement: Supplementary file 5 — Additional file 5: Fig. S5. Proteomic analysis of lysates from Trem2 KO and WT microglia (continued). (A) Effects of Trem2 deletion on DAM-responsive proteins with tau or LPS/ATP treatment. Log2 fold-change of DAM-associated DEPs identified in pairwise comparisons from run 1 (tau) or run 2 (LPS/ATP, gray) and adj p-values are indicated in the left heatmaps. Z-score heatmaps depict calculated z-scores of 6 replicates from run 1 (left) and run 2 (right) are shown on the adjacent heatmaps to the right; “NA” values derived from missing peptide values are indicated in gray. (B,C) Overlapping DEPs in response to both tau and LPS/ATP in WT (B) and Trem2 KO (C) microglia are depicted in the Venn diagrams, and Log2 fold-change, adj p-values and z-scores of the 302 WT (B) or 473 KO (C) DEPs are plotted in the heatmaps indicated. Overlapping WT (B) and KO (C) DEPs were also plotted for Log2 fold-change distribution in the bottom scatterplots for change in response to tau (x-axis) and LPS/ATP (y-axis). [file 13024_2022_562_MOESM5_ESM.jpg]

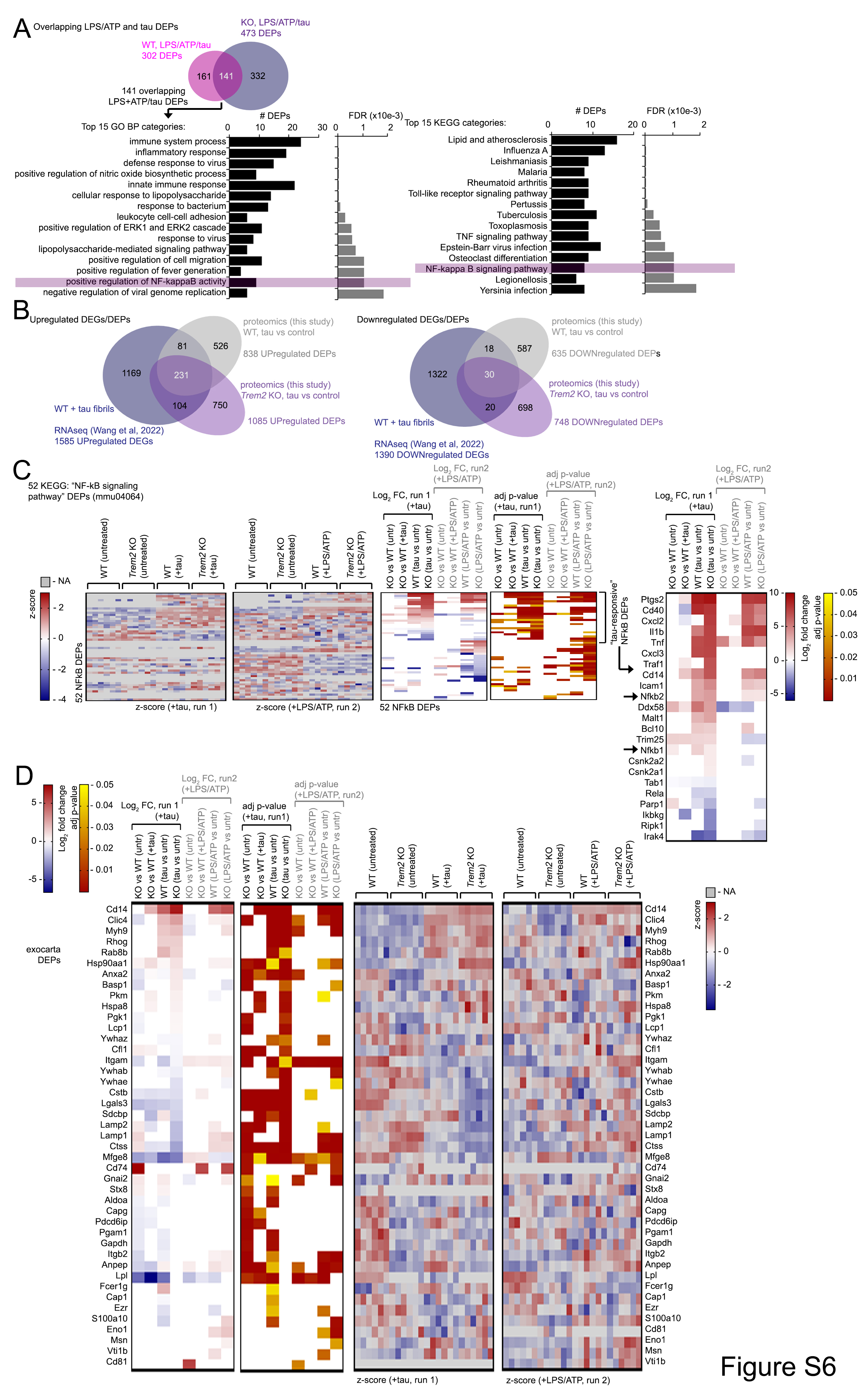

Supplement: Supplementary file 6 — Additional file 6: Fig. S6. Effects of Trem2 deletion on proteomic changes with tau and LPS/ATP treatment (continued). (A) Gene Ontology (GO DAVID) analysis of 141 overlapping genes that change in response to tau and LPS/ATP in both WT and KO microglia. Number of DEPs and FDR for the top 15 GO BP (left graphs) and KEGG categories (right graphs) are shown. NFkB categories in BP and KEGG analysis are highlighted in purple. (B) Overlap in DEPs identified in WT (gray) and Trem2 KO (purple) response to tau oligomers (proteomics, this study), and DEGs identified in WT microglia treated with tau fibrils [62] (violet) by RNAseq analysis. (C) Z-scores for 6 replicates from run 1 (left) and run 2 (right) are shown on the left, “NA” values derived from missing peptide values are indicated in gray. Adjacent heatmaps depicting Log2 fold-change and adj p-values of 52 DEPs identified by GO KEGG analysis; Log2 fold-change heatmaps for “tau-responsive” NFkB DEPs (from run 1) are also shown, Nfkb1 and 2 are indicated by black arrows. (D) Heatmaps depicting Log2 fold-change and adj p-values of potential exosome-associated DEPs in the mouse microglia Exocarta database. Z-scores for 6 replicates from run 1 (left) and run 2 (right) are shown on the adjacent heatmaps to the right, “NA” values derived from missing peptide values are indicated in gray. [file 13024_2022_562_MOESM6_ESM.jpg]

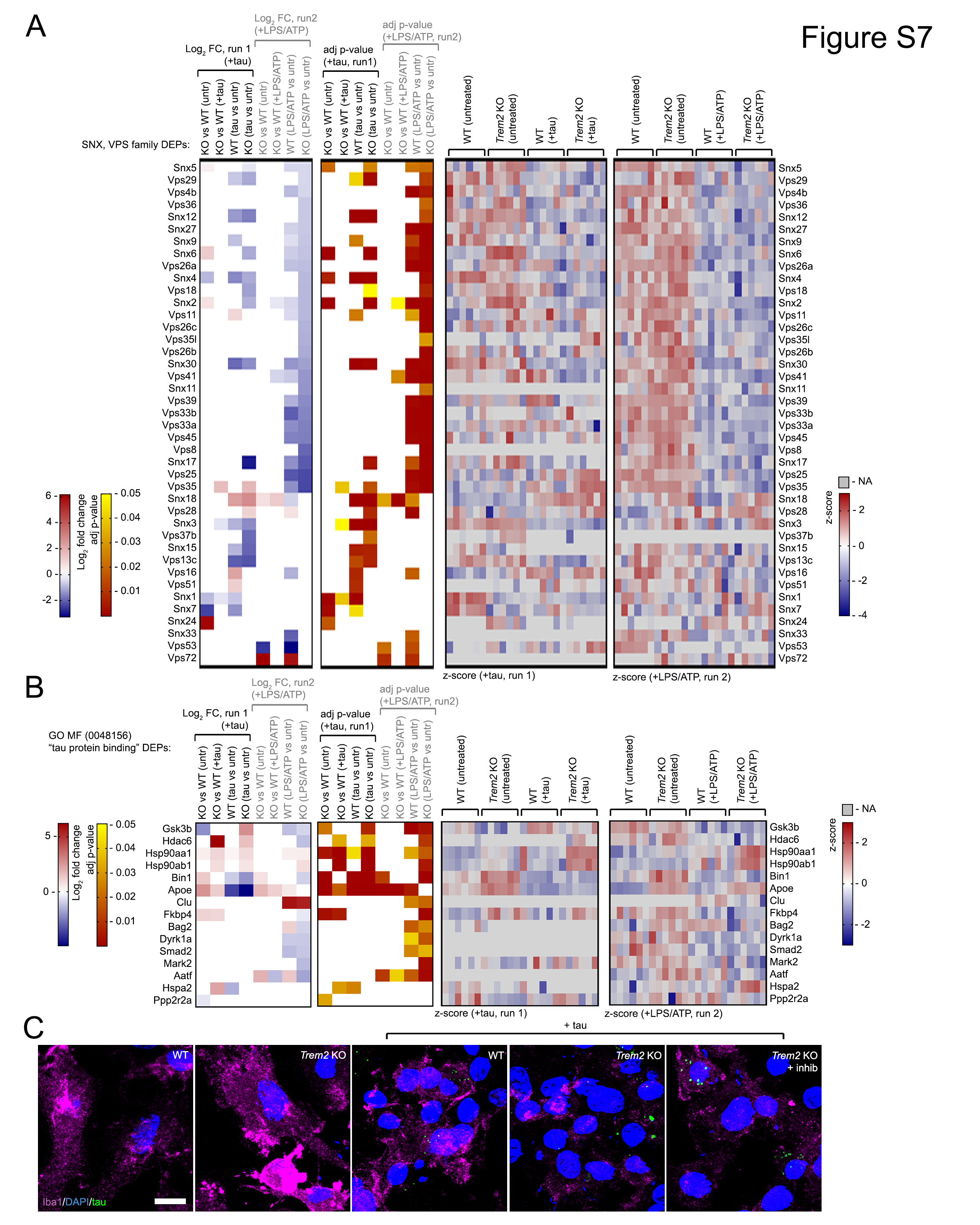

Supplement: Supplementary file 7 — Additional file 7: Fig. S7. Effects of Trem2 deletion on proteomic changes with tau and LPS/ATP treatment (continued). (A) Heatmaps depicting Log2 fold-change and adj p-values of SNX and VPS DEPs by pairwise comparison of the treatment groups indicated in runs 1 and 2. Z-scores for 6 replicates from run 1 (left) and run 2 (right) are shown on the adjacent heatmaps to the right, “NA” values derived from missing peptide values are indicated in gray. (B) Heatmaps depicting Log2 fold-change and adj p-values of GO MF “tau protein binding” DEPs by pairwise comparison of the treatment groups indicated in runs 1 and 2. Z-scores for 6 replicates from run 1 (left) and run 2 (right) are shown on the adjacent heatmaps to the right, “NA” values derived from missing peptide values are indicated in gray. (C) Representative images of tau loading (2.5 μg/ml, 24 h) in WT and Trem2 KO microglia with and without tau and GW4869 (inhib). Cells were fixed and stained to visualize Iba1 (purple), tau (green) and nuclei (DAPI, blue) as indicated. Bar = 10 μm. [file 13024_2022_562_MOESM7_ESM.jpg]
